# Intrasplenic Thymus Organogenesis from Injectable Tissue Fragments Restores Functional T‐Cell Immunity

**DOI:** 10.1002/advs.77358

**Published:** 2026-08-25

**Authors:** Shaocong Wang, Zijian Zhang, Fulin Dai, Zhijie Yin, Zhen Xing, Yurong Li, Haochong Lei, Junfeng Zhang, Chunyan Liu, Yan Li, Lei Dong

**Affiliations:** ^1^ State Key Laboratory of Pharmaceutical Biotechnology School of Life Sciences Nanjing University Nanjing Jiangsu China; ^2^ National Resource Center for Mutant Mice MOE Key Laboratory of Model Animal for Disease Study Model Animal Research Center Department of Oncology Nanjing Drum Tower Hospital Affiliated Hospital of Medical School Nanjing University Nanjing China; ^3^ Wuxi Xishan Institute of Applied Biotechnology Nanjing University Wuxi Jiangsu China; ^4^ Jiangsu Key Laboratory of Sericultural Biology and Biotechnology School of Biotechnology Jiangsu University of Science and Technology Zhenjiang Jiangsu China; ^5^ Faculty of Life Sciences & Medicine King's College London London UK; ^6^ Chemistry and Biomedicine Innovation Center (ChemBIC) ChemBioMed Interdisciplinary Research Center at Nanjing University Nanjing Jiangsu China

**Keywords:** ectopic regeneration, immunodeficiency, spleen, supportive niche, thymus, T‐cell immunity

## Abstract

Thymic hypofunction due to primary defects, aging, infection, or cytoreductive therapies causes profound restriction of the T‐cell receptor (TCR) repertoire and increases rates of morbidity and mortality. Restoring thymic function is therefore critical. Here, the distinctive niche within the spleen has inspired the development of intrasplenic thymus regeneration (ISTR), an approach in which cultured thymic tissue fragments are implanted into the spleens of athymic nude mice, aged mice, thymectomized mice undergoing hematopoietic stem cell transplantation (HSCT), and humanized mice. The spleen's abundant vasculature and supportive stromal milieu enable rapid thymic organogenesis, leading to the reconstitution of phenotypically diverse and functional T‐cell compartments, as well as protective responses to viral and tumor challenges. Relative to intramuscular implantation, ISTR drives faster and more complete thymic development, generates greater T‐cell output, and is associated with a lower incidence of graft‐vs.‐host disease. In humanized animals, intrasplenic thymus grafts also promote the rapid emergence of functional human T cells. These results identify the spleen as an optimal ectopic niche for thymus regeneration and provide a promising strategy for clinical immune reconstitution and regenerative immunology.

## Graphical abstract

Clinical intramuscular thymus transplantation yields only short‐lived efficacy and marginal therapeutic benefits. Benefiting from the spleen's intrinsic strengths—rapid vascular perfusion, abundant developmental factors, and resident progenitors—the intrasplenic thymic grafts achieve robust thymic regeneration and substantial T‐cell reconstitution compared with intramuscular grafts. This strategy ultimately restores fully functional T‐cell immunity while lowering the incidence of graft‐vs.‐host disease.

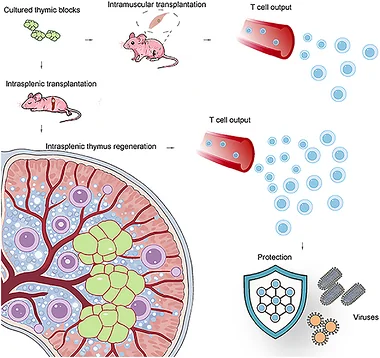

## 1 Introduction

The thymus is the primary lymphoid organ for T‐cell development and the establishment of immune tolerance [1, 2, 3, 4]. Its stromal network provides a specialized microenvironment for thymocyte differentiation [5]. However, the thymus is highly susceptible to insults and involution. Aging, cytoreductive therapies, infections, steroid hormones, immunosuppressive drugs, and other exogenous stresses can lead to thymic atrophy, resulting in a reduction of thymic mass, disruption of its architecture, and a sharp decline in the output of naïve T cells [6, 7]. Congenital thymus defects are commonly accompanied by profound immune disorders. Thymectomy is routinely performed during pediatric cardiac surgery; it was reported that all‐cause mortality and the risk of cancer increased among patients who had undergone thymectomy [8]. In hematopoietic stem cell transplantation (HSCT), a vital treatment for various hematological disorders, post‐transplant T‐cell deficiency is a major contributor to morbidity and mortality [9, 10, 11, 12, 13]. In summary, thymic loss or degeneration leads to diminished naïve T‐cell output and a narrowed TCR repertoire. These deficits, in turn, impair adaptive immune function, increasing susceptibility to infections, autoimmunity, cancer, and impaired vaccine responses [6].

Conventional therapeutic strategies for promoting thymic regeneration primarily involve hormonal modulation, cytokine administration, and nutritional interventions to induce endogenous thymic regeneration. These include growth hormone pathway agonists (keratinocyte growth factor (KGF), growth hormone (GH), Thymosin‐α1), interleukin‐7 (IL‐7), and sex steroid ablation [14], all of which have been demonstrated to transiently enhance thymopoiesis in preclinical models and clinical trials; however, sustained improvement remains difficult to achieve. Other potential therapies include cellular therapies (progenitor cells, epithelial cells, endothelial cells, and mesenchymal cells), gene therapy [15, 16], and tissue engineering of thymic scaffolds or organoids [17, 18, 19]. Nevertheless, most of these therapies can be futile when the thymus is completely deficient, irreversibly dysfunctional, or removed. Thymic organoids are still unable to fully recapitulate the architectural complexity and physiological function of the intact, in vivo thymus. Thus, thymus transplantation remains the sole curative therapy in specific clinical contexts. This is best exemplified by congenital athymia, a condition in which infants are unable to generate T cells. If untreated, this disorder is invariably fatal, with most patients succumbing to infection by two years of age [20].

Allogeneic thymus tissue transplantation has been performed in infants with complete DiGeorge syndrome [21]. Transplanted cultured thymus in the quadriceps muscle showed evidence of thymopoiesis within 5–6 months [22] or after a year [23]. Only ∼75% of infants survived long term with T‐cell counts below the 10th percentile for age, and immune recovery took months or even a year in survivors [22, 24, 25]. Mortality has largely been attributed to incomplete engraftment and/or delayed immune reconstitution [26]. Heart‐thymus en bloc transplantation has been tested in nonhuman primates [27]. Yet, this approach is exclusively indicated for patients requiring concomitant heart and thymus transplantation and is only feasible with fresh thymus and requires technically formidable procedures. Overall, current thymus‐regenerative interventions remain limited in both efficiency and kinetics, and their beneficial effects are often temporary. These limitations underscore the need for alternative implantation strategies that could better support thymic regeneration.

We notice that the architecture, cellular composition, and microenvironment of the spleen exhibit a remarkable alignment with the essential drivers of thymus regeneration and propose the spleen as an innovative and promising site for thymic regeneration. The spleen is a highly vascular, loosely structured secondary lymphoid organ that can naturally support the survival of a variety of immune cells (T and B lymphocytes, dendritic cells (DCs), macrophages). Furthermore, the spleen itself possesses intrinsic mechanisms for inducing systemic immune tolerance maintained by splenic DCs [28], macrophages [29], B‐cell subtypes [30], and regulatory T cells (Tregs) [31, 32]; and the splenic stroma includes mesenchymal elements that can secrete extracellular matrix and trophic factors [33, 34]. These features indicate that the spleen may provide a fertile milieu of signals, modulators, and vascular nourishment conducive to lymphoid tissue engraftment.

Notably, recent work has demonstrated that the spleen can act as an in vivo “bioreactor” for organ growth. For example, after implantation of hepatocytes with a tissue matrix extract, Wang et al. demonstrated that the spleen‐supported grafts developed into vascularized, functional liver‐like tissue without immune rejection [35]. The results indicate that the spleen's environment is not only permissive to allogeneic cell engraftment but may even promote regeneration with reduced host response. Such findings validate that the spleen's structure, blood supply, and immunological network can safely sustain ectopic tissue growth.

In addition, the spleen naturally participates in hematopoiesis during stress by expanding hematopoietic progenitors [36, 37]. Therefore, we aim to leverage the spleen's advantages of rich blood supply and abundant resident immune and stromal cells to provide a trophic and supportive niche for implanted thymic epithelial cells and “nurse” the development of thymocytes.

Here, we tested our ISTR strategy for its ability to drive thymus development and enhance functional competence by introducing ex vivo‐cultured or cryopreserved thymic tissue into the splenic parenchyma. Notably, this strategy enables substantial reconstruction of diverse T‐cell compartments in both nude and humanized mouse models with normal phenotypes and functions. In this paper, we report the discovery of a novel, nutrient‐rich niche within the spleen that exhibits remarkable fitness for supporting thymus regeneration.

## 2 Results

### 2.1 Efficient Ectopic Thymus Regeneration in the Spleen

To assess thymic regenerative potential in the splenic niche, we transplanted GFP‐labeled neonatal C57BL/6J thymic tissue into athymic BALB/c nude mice, modeling allogeneic thymus transplantation. Fresh grafts caused severe graft‐vs.‐host disease (GVHD) within 4–6 weeks, including diarrhea, rectal prolapse, skin thinning, wasting, and weight loss (Figure S1A). We therefore cultured thymic tissue in vitro before transplantation to deplete donor thymocytes. After ≥6 days of culture, tissues changed from lymphoid‐rich to epithelial‐dominant morphology, while CK5^+^ or CK8^+^ single‐positive and CK5^+^CK8^+^ double‐positive thymic epithelial cell (TEC) populations were preserved (Figure S1B–D). CD3^+^ thymocytes were efficiently removed, and flow cytometry further showed near‐complete depletion of dendritic cells and B cells (Figure S1E–G). Thus, cultured, stromal cell‐enriched thymic tissue was used for subsequent experiments.

**FIGURE 1:**
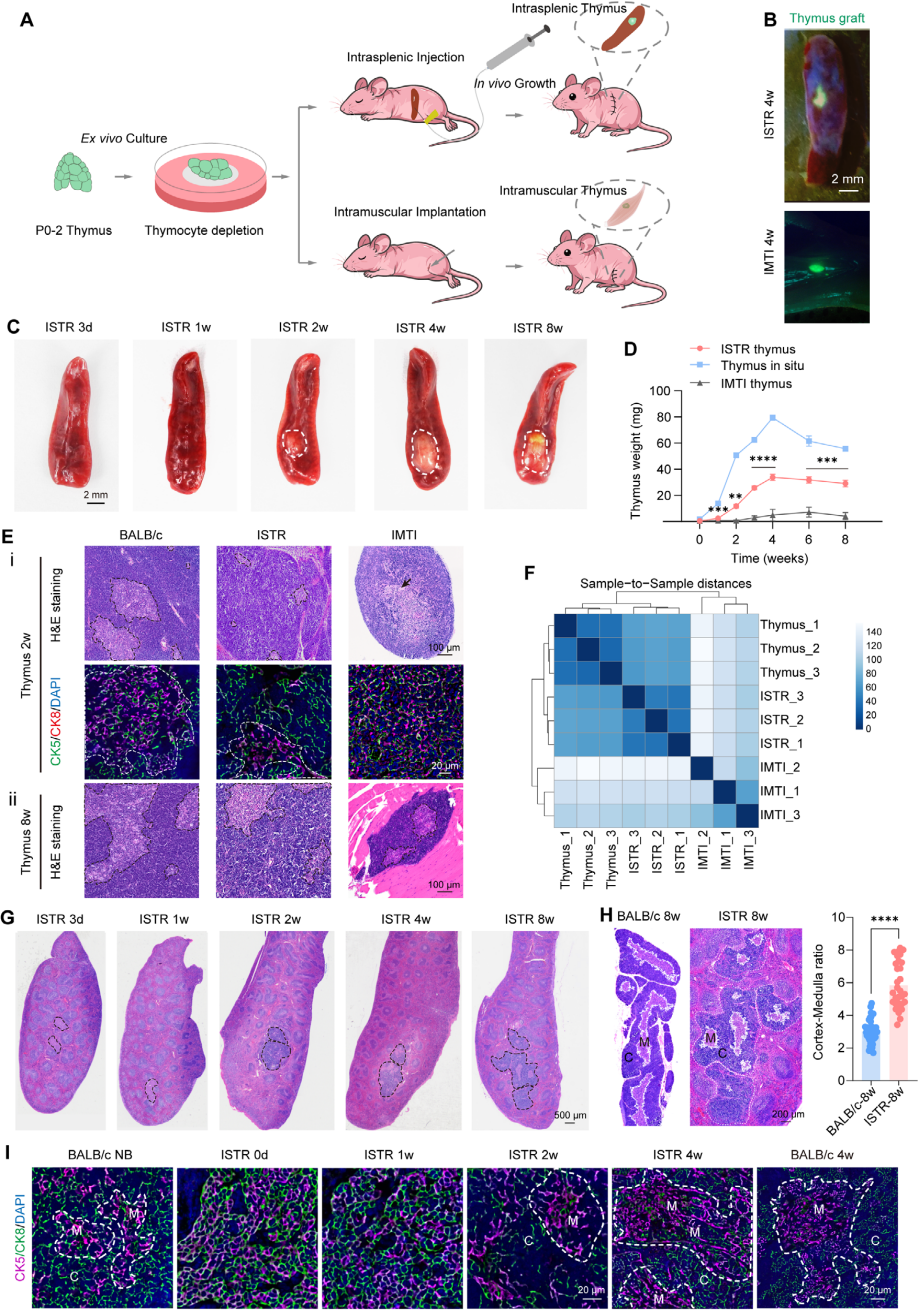
Intrasplenic transplantation of cultured thymic fragments yields organized thymic architecture. (A) Experimental schematic. Neonatal GFP‐transgenic thymic lobes were cultured ex vivo for 7 days and then delivered into the spleens or quadriceps femoris of 4‐week‐old nude mice via a 20‐G needle (graft size ∼0.7 mm). (B) Fluorescence imaging of recipient spleens or quadriceps femoris 4 weeks post‐transplantation under GFP excitation. Scale bar: 2 mm. (C) Macroscopic appearance of spleens at indicated time points after grafting. Scale bar: 2 mm. (D) Temporal changes in graft mass from week 0 to week 8 post‐transplantation (n = 5 per time point). Pink line: weight of intrasplenic thymus grafts from nude recipients post‐graft. Gray line: weight of intramuscular thymus graft from nude recipients post‐graft. Blue line: weight of wild‐type BALB/c thymus collected at the same post‐transplantation interval as recipient cohorts. (E) Representative morphology of thymic grafts from the spleen or quadriceps femoris at weeks 2 and 8, compared with endogenous thymus from tissue‐age‐matched (2‐week‐old or 8‐week‐old) BALB/c mice. i: Week 2 graft histology: H&E staining (top) and immunofluorescence for CK8 (cortex) and CK5 (medulla) in splenic and muscular grafts (bottom). Scale bars: 100, 20 µm. ii: Week 8 graft histology: H&E staining of intrasplenic vs. intramuscular thymic tissue. Scale bars: 100 µm. (F) Sample‐to‐sample distances of transcripts of intrasplenic thymus grafts (two weeks post‐graft), intramuscular thymus grafts (two weeks post‐graft), and two‐week‐aged BALB/c thymus. (G) H&E staining of intrasplenic grafts at days 3, 7, 14, 28, and 56. Scale bar: 500 µm. (H) Left: representative gross morphology of thymic grafts at week 8, compared with the endogenous thymus of age‐matched (8‐week‐old) BALB/c mice. Scale bar: 5 mm. Right: cortical‐to‐medullary area ratio of week 8 grafts, measured from H&E sections using ImageJ (n = 5). Scale bar: 200 µm. (I) Immunofluorescence for cytokeratin 8 (CK8; cortex) and cytokeratin 5 (CK5; medulla) in native and intrasplenic thymic tissue. Scale bars: 20 µm. Data are shown as mean ± SEM. Statistical comparisons were made by two‐way ANOVA (D) or unpaired two‐tailed Student's *t*‐test [(H)]; ^*^ *p* < 0.05, ^****^ *p* < 0.0001, n.s., not significant. All experiments were performed in three independent cohorts, except panel D.

We optimized intrasplenic thymus transplantation (ISTR) by injecting equal‐volume, equal‐mass cultured fragments (0.2–0.9 mm) into the splenic parenchyma (Figure 1A). At week 4, graft weight and structural integrity increased with fragment diameter up to 0.7 mm, with no further benefit at 0.9 mm (Figure S1H, I). Because 0.9‐mm grafts increased hemorrhage risk, 0.7‐mm fragments were selected. We then compared ISTR with intramuscular thymus implantation (IMTI) in the quadriceps using matched grafts (Figure 1A). GFP imaging showed larger ISTR grafts at week 4 (Figure 1B). Although grafts at both sites grew progressively and peaked at weeks 4–6, ISTR grafts reached ∼50% of the age‐matched BALB/c thymus weight by week 8, whereas IMTI grafts remained below 10% (Figure 1C, D and Figure S2). Differences were evident by week 2. At this time, splenic grafts were larger, lymphoid‐like, and displayed distinct CK8^+^ cortical and CK5^+^ medullary compartments; muscle grafts remained epithelial‐like, poorly organized, and partially necrotic (Figure 1E, i). By week 8, IMTI grafts also formed cortico‐medullary structures but remained markedly smaller (Figure 1E, ii). Transcriptomic profiling at week 2 further placed ISTR grafts closer to the native BALB/c thymus than to IMTI grafts (Figure 1F), indicating that the spleen's environment accelerates thymic development.

The spleen's open circulation and higher hemoglobin content may favor early delivery of oxygen and nutrients (Figure S3A). However, comparable graft vessel density at week 2 suggested that vascularization was not the principal determinant of site‐specific development (Figure S3B). To define niche‐specific mechanisms, we profiled spleen and quadriceps tissues from 4‐week‐old nude mice and native BALB/c thymus based on known thymopoietic factors. Transcriptome analysis showed that the spleen more closely resembled the thymus than muscle in the expression of thymopoietic factors, including *Ptma, Ltb, Tnfsf11*, and *Flt3l* (Figure S3C). *Lta, Gh, Igf1, Kitl*, and *Il12a* were highly expressed in the spleen, which can promote both TEC regeneration and thymocyte maturation, whereas muscle preferentially expressed only *Fgf7* and *Bmp4*, which can only facilitate TEC regeneration. Overall, thymopoietic factors were enriched in the spleen (Figure S3C,D). Given the bidirectional crosstalk required between thymocytes and thymic epithelial cells for proper thymus development [38], the spleen may offer a more supportive microenvironment. In addition, thymic regeneration relies on precursor cell quantity and quality [39, 40, 41, 42]. Results showed that common lymphoid progenitors (CLP) were more abundant in the spleen than in muscle, without significant differences in lymphoid‐primed multipotent progenitors (LMPP) (Figure S3E–G). These findings suggest that the splenic niche promotes regeneration through coordinated thymopoietic signals and increased lymphoid progenitor availability.

We next characterized ISTR graft maturation. Hematoxylin and eosin (H&E) staining showed progressive conversion from epithelial‐like tissue to organized lymphoid structures, with cortico‐medullary differentiation evident by week 2 (Figure 1G). By week 8, cortical‐to‐medullary area ratios were comparable to those of native BALB/c thymus (Figure 1H). CK5/CK8 staining showed re‐establishment of cortical and medullary compartments and progressive expansion of medullary islets after transplantation (Figure 1I). Vascularization began by day 3, formed robust host‐graft connections by week 1, and matured thereafter (Figure S4A,B). Grafts also showed appropriate medullary enrichment of dendritic cells and MHC‐II^+^ cells, as well as AIRE^+^ and involucrin^+^ medullary epithelial cells (Figure S4C,D). At week 8, *Foxn1*, *Ccl25*, and *Dll4* expression did not differ significantly from native thymus (Figure S4E). Variability in *Dll1* and *Il7* expression may reflect residual surrounding tissue during graft dissection. Moreover, grafts progressively acquired blood‐thymus barrier features, including reduced dextran permeability, capsular collagen deposition, and continuous claudin‐5^+^ endothelial tight junctions (Figure S5A–C). Together, these data demonstrate intact developmental progression and compartmental autonomy within the tissue.

Collectively, cultured thymic tissue undergoes organotypic regeneration in the spleen, restoring cortico‐medullary architecture, specialized epithelial and stromal populations, vascular integration, and barrier properties. Compared with muscle, the spleen provides a more favorable niche for thymic growth and maturation, supporting further evaluation of ISTR as a potential strategy for thymus regeneration.

### 2.2 T‐Cell Reconstitution in ISTR Mice

**FIGURE 2:**
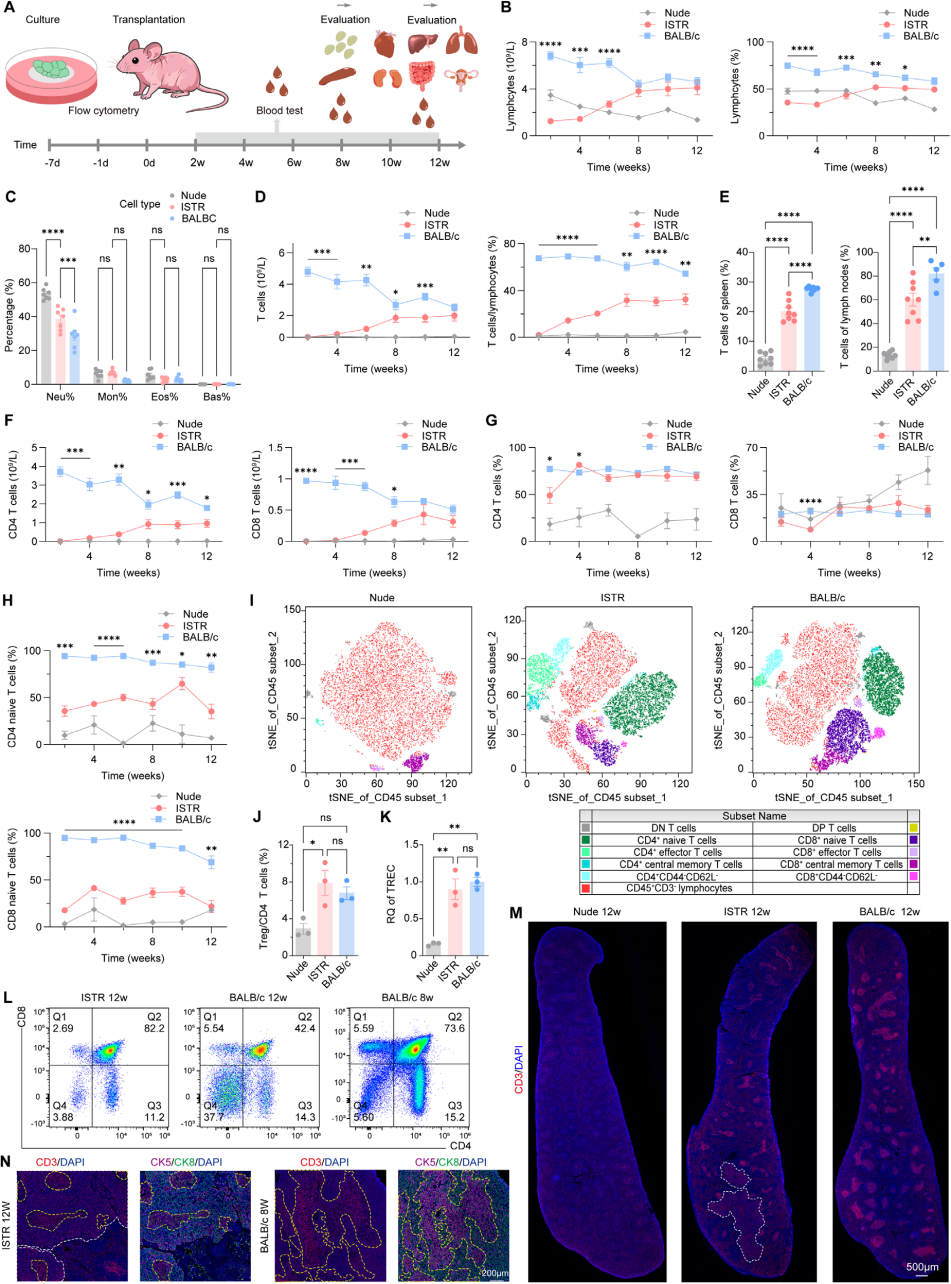
Intrasplenic thymic grafts rapidly reconstitute a diverse peripheral T‐cell compartment. (A) Experimental timeline: nude mice received intrasplenic thymus grafts, with peripheral blood sampling every two weeks; terminal analyses were performed at week 8 and week 12 post‐transplantation. (B) Absolute counts and percentages of total lymphocytes in peripheral blood from age‐matched nude (gray), ISTR (red), and BALB/c (blue) mice (n = 7, 6–16 weeks old). (C) White blood cell differentials at week 8 post‐transplant, determined by automated hematology (n = 7). (D) Absolute numbers and percentages of CD3^+^ T cells in peripheral blood over time (n = 7). (E) Proportions of CD3^+^ T cells in spleen and lymph nodes at week 8 (in spleen, n = 8; in lymph nodes, n = 8 for nude and ISTR groups, n = 5 for BALB/c group). (F, G) Absolute counts (F) and percentages (G) of CD4^+^ and CD8^+^ T‐cell subsets among total T cells in peripheral blood at various time points (n = 7). (H) Frequencies of naïve (CD62L^+^CD44^−^) CD4^+^ and CD8^+^ T cells in peripheral blood over time (n = 7). (I) t‐SNE projection of major lymphocyte subsets in peripheral blood at week 8. (J) Frequency of FoxP3^+^ regulatory T cells among CD4^+^ T cells at week 8 (n = 3). (K) Relative quantification of TRECs by qPCR in PBMC at week 8 (n = 3). (L) Proportions of CD4^+^CD8^−^ (SP), CD4^−^CD8^+^ (SP), and CD4^+^CD8^+^ (DP) thymocytes in ISTR grafts at week 8 compared to native thymus from week 8 and week 12 BALB/c mice (n = 5). (M) Immunofluorescence of CD3^+^ cells in spleen sections at week 8; graft boundary indicated by white dashed line. Scale bar: 500 µm. (N) Co‐localization of CD3dim cortical (CK8^+^) and CD3high medullary (CK5^+^) regions in ISTR and native thymus by dual immunofluorescence. Scale bar: 200 µm. Data are mean ± SEM. Statistical analyses: one‐way ANOVA [(C), (E), (J) and (K)] or two‐way ANOVA [(B), (D), (F) to (H)]; ^*^ *p* < 0.05, ^****^ *p* < 0.0001; n.s., not significant. All experiments were independently repeated three times per group, except panels K and L.

Here, we evaluated the capacity of intrasplenic thymus grafts to support T‐cell maturation in BALB/c nude mice, which lack an endogenous thymus and mature T cells. Successful thymic regeneration should restore peripheral T‐cell compartments comparable to those of immunocompetent BALB/c mice. Preliminary assessment confirmed that the transplantation procedure itself caused no detectable physiological perturbation (Figure S6A,B). Three cohorts were therefore established: untreated nude mice (Nude), nude mice with ISTR, and age‐matched immunocompetent BALB/c mice (BALB/c), followed longitudinally for 12 weeks after transplantation (Figure 2A).

Hematological analysis revealed progressive restoration of lymphocyte populations in ISTR mice, with both absolute numbers and percentages increasing over time and approaching BALB/c levels (Figure 2B). At week 8, monocyte, eosinophil, and basophil populations were comparable among groups, whereas neutrophil counts in ISTR mice gradually shifted from the elevated nude baseline toward BALB/c ranges, suggesting T‐cell reconstitution without major disruption of innate immunity (Figure 2C). Flow‐cytometric analysis demonstrated progressive recovery of peripheral CD3^+^ T cells in ISTR mice, which became detectable at week 2 and plateaued by week 8, reaching approximately 50% of BALB/c levels (Figure 2D and Figure S6C). T‐cell frequencies in secondary lymphoid organs were significantly increased compared with nude mice, with some ISTR mice approaching BALB/c levels (Figure 2E). CD4^+^ T‐cell numbers in PBL stabilized at approximately 50% of BALB/c levels, whereas CD8^+^ T‐cell numbers showed no significant difference between ISTR and BALB/c mice after 8–10 weeks (Figure 2F). Importantly, the CD4/CD8 ratio in ISTR mice resembled that of BALB/c mice (60%–80% CD4^+^ vs. 20%–30% CD8^+^) from week 4 onward, in contrast to the CD8^+^ predominance observed in nude controls (Figure 2G) [43]. Naïve T‐cell frequencies (CD62L^+^CD44^−^) in ISTR mice remained intermediate between nude and BALB/c groups (Figure 2H and Figure S6C).

We also quantified peripheral reconstitution in IMTI recipients at 8 weeks post‐graft, a time point at which ISTR mice attained a near steady state. Compared with IMTI, ISTR mice exhibited higher lymphocyte levels and lower neutrophil proportions at week 8, despite similar total white blood cell counts (Figure S7A), indicating a more favorable immune reconstruction profile. Consistently, ISTR mice showed significantly higher total T‐cell numbers, CD4^+^/CD8^+^ T‐cell populations, and naïve T‐cell frequencies than IMTI mice, demonstrating superior T‐cell output and quality after splenic thymus transplantation (Figure S7B–D).

High‐dimensional t‐SNE analysis further confirmed broad recovery of T‐cell subsets in ISTR mice, with immune profiles closely resembling BALB/c controls except for modestly reduced naïve populations (Figure 2I). The marked enrichment of αβ T cells indicated intact thymic differentiation. Other lymphocyte populations, including B cells and NK cells, as well as myeloid populations within the lymphocyte gate, were also detected (Figure S8). Regulatory T‐cell frequencies within splenic CD4^+^ compartments were comparable between ISTR and BALB/c mice (Figure 2J and Figure S9A,B), suggesting preservation of central tolerance. T‐cell receptor excision circle (TREC) analysis further demonstrated predominant thymic‐derived T‐cell production in ISTR mice, in contrast to nude controls (Figure 2K).

Interestingly, ISTR mice exhibited increased frequencies of CD4^+^CD8^+^ double‐positive (DP) thymocytes at 12 weeks compared with age‐matched BALB/c mice, resembling the profile of the younger 8‐week‐old thymus (Figure 2L and Figure S10), suggesting that the regenerated thymus retained donor‐derived developmental characteristics independent of host aging [44]. Histological analysis revealed restoration of organized periarteriolar lymphoid sheaths in ISTR spleens, contrasting with the sparse and disorganized CD3^+^ T‐cell distribution in nude mice (Figure 2M). Within the graft region, CD3^dim^ cortical thymocytes surrounded CD3^high^ medullary populations with clear cortico‐medullary organization, indicating ordered thymic maturation (Figure 2N).

We next examined the functional competence of regenerated T cells. In lymphocyte transformation assays, ISTR mice exhibited comparable Con A‐induced proliferation responses to BALB/c controls, as assessed by CFSE dilution (Figure S11A,B). In antigen‐specific assays, B16‐OVA‐immunized ISTR mice mounted detectable OVA‐specific T cell responses upon ex vivo restimulation with full‐length OVA protein, whereas nude mice showed negligible activity. ELISPOT analysis demonstrated that ISTR‐derived T cells achieved more than 50% of BALB/c T‐cell activity in response to OVA stimulation (Figure S11C–E), confirming restoration of antigen‐specific immunity.

To define MHC restriction of regenerated T cells, we performed T‐cell–DC co‐culture assays using antigen‐presenting cells with different MHC backgrounds. After OVA immunization, responder T cells from ISTR and BALB/c mice were stimulated with donor (C57BL/6J, H‐2^b^) or recipient (BALB/c, H‐2^d^) DCs, with or without OVA loading. BALB/c T cells responded strongly to OVA‐loaded BALB/c DCs and exhibited alloreactivity toward B6 DCs. Notably, ISTR‐derived T cells were activated by OVA‐loaded BALB/c DCs, demonstrating recognition of antigen presented by recipient MHC, consistent with in vivo infection results. ISTR T cells also retained measurable reactivity toward B6 DCs, and stronger activation by OVA‐loaded B6 DCs suggested the presence of antigen‐specific clones capable of responding to B6 MHC presentation (Figures S12 and S13).

Together, these results demonstrate that ISTR enables robust reconstruction of a functional and tolerant T‐cell compartment, providing more efficient and safer immune reconstitution than conventional thymus transplantation approaches.

### 2.3 T‐Cell Receptor Repertoire Diversity and GVHD Assessment in ISTR Mice

**FIGURE 3:**
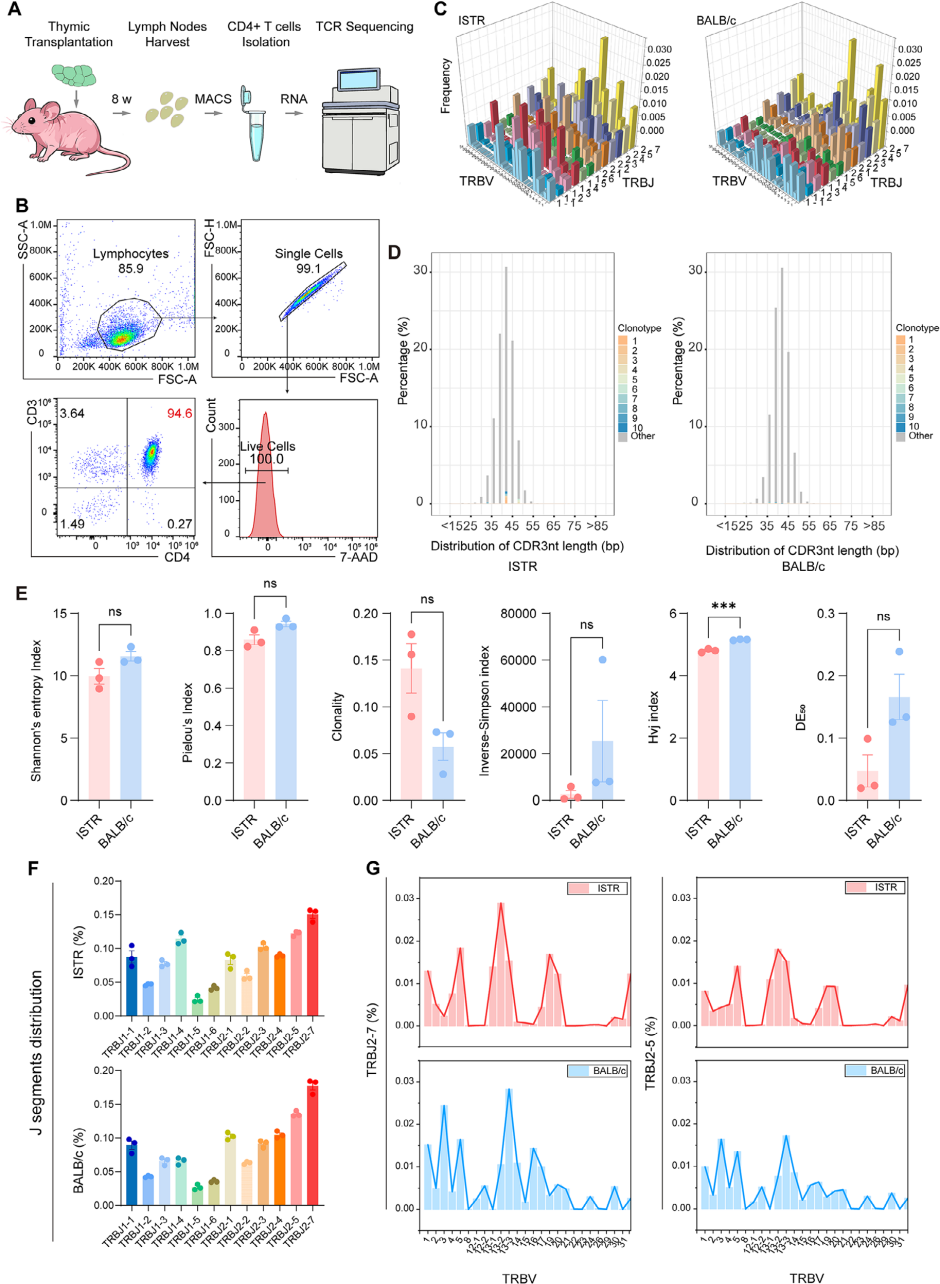
TCR repertoire diversity in ISTR mice by high‐throughput sequencing of the Vβ chain of lymph node‐isolated CD4^+^ T cells. (A) Experimental scheme: cultured thymic tissue was implanted to the spleen 8 weeks prior to lymph node isolation; CD4^+^ T cells were sorted, and RNA was extracted and sequenced to assess the TCR repertoire. (B) Determination of the enriched CD4^+^T cells by flow cytometry and the gating strategy. (C) Distribution of TCRβ V‐J pairs in ISTR vs. 12‐week‐old BALB/c mice shown as 3D bar graphs. (D) CDR3 length distribution in ISTR mice and BALB/c mice. (E) Indices of TCR diversity based on the frequencies of TCR clones of ISTR and BALB/c mice of the same age. (F, G) TCR clonal distribution of J region (F) and V‐region distribution within the top 2 abundant J regions (G). In experiments for each group (n = 3), data are represented as mean ± SEM. Statistical analysis was performed using unpaired two‐tailed Student's *t*‐tests for panel E. ^*^ *p* < 0.05, ^****^ *p* < 0.0001, n.s., not significant.

Emerging evidence indicates that thymic function and CD4^+^ T‐cell reconstitution are closely associated with transplant outcomes, including survival, GVHD development, and immune susceptibility. To characterize the quality and diversity of regenerated T cells, we performed TCR sequencing of CD4^+^ T cells isolated from major lymph nodes (cervical, axillary, brachial, abdominal, inguinal, and mesenteric lymph nodes) of ISTR mice at 8 weeks post‐grafting and age‐matched BALB/c controls (Figure 3A,B). Similar to BALB/c mice, ISTR mice exhibited extensive TCR clonal diversity, as demonstrated by comparable V‐J pairing distributions and three‐dimensional TCR clonotype landscapes (Figure 3C). The CDR3 length distributions displayed Gaussian‐like patterns in both groups, indicating a non‐skewed TCR repertoire associated with immune homeostasis (Figure 3D).

To further quantify repertoire diversity and clonotype evenness, we analyzed Shannon entropy, Pielou's index, clonality, inverse Simpson diversity, Hvj index, and DE50 (Equations S1–S6). Although several indices showed a slight reduction in diversity in ISTR mice, no significant differences were observed except for the Hvj index (Figure 3E). Moreover, the distribution of J segments and dominant V‐J combinations was highly similar between ISTR and BALB/c mice (Figure 3F,G). These results demonstrate that intrasplenic thymus grafts restore a broad and balanced TCR repertoire comparable to that of immunocompetent mice.

Beyond peripheral T‐cell reconstruction, we evaluated the potential risk of GVHD following ISTR. During the 12‐week observation period, ISTR mice displayed no apparent abnormalities in gross morphology or tissue histology (Figure S14A–C). Serum IFN‐γ levels were comparable among nude, ISTR, and BALB/c groups, while TNF‐α levels showed no difference between nude and ISTR mice, although they remained higher than BALB/c controls (Figure S14D). Consistently, serum IgG/IgM levels, hematological parameters, and liver function markers (ALT and AST) revealed no evidence of systemic inflammation or organ injury (Figure 2C and Figure S14E,F). Comprehensive GVHD scoring across independent cohorts further demonstrated that ISTR mice exhibited significantly reduced wasting symptoms and higher body weight compared with IMTI mice (Figure S14G–I). Accordingly, both GVHD incidence and severity were markedly decreased following ISTR transplantation, supporting improved safety compared with conventional intramuscular thymus transplantation.

To further assess long‐term immune tolerance, we examined 10‐month‐old ISTR mice together with age‐matched BALB/c and nude controls. No overt phenotypic abnormalities were observed in ISTR mice (Figure S15A). Although some ISTR mice developed mild GVHD‐like manifestations, including mild diarrhea, rectal prolapse, and hunched posture (Figure S15B), peripheral T‐cell composition remained comparable to that of BALB/c mice, including total T cells, CD4^+^ and CD8^+^ T cells, and Tregs, whereas nude mice exhibited profound T‐cell deficiency (Figure S15C–E). A subset of ISTR mice showed increased PD‐1^+^ T‐cell frequencies without elevated CD69 expression (Figure S15F,G), suggesting the establishment of a modest inhibitory adaptation in response to persistent low‐level alloantigen exposure. Histological analyses revealed only mild diffuse lymphocytic infiltration in the spleen and liver of some ISTR mice (Figure S15H). Importantly, no significant fibrosis was detected in liver or lung tissues, and no skin manifestations characteristic of cGVHD, including epidermal atrophy, collagen deposition, adnexal destruction, or dermal lymphocyte infiltration, were observed (Figure S15I).

Together, these findings demonstrate that intrasplenic thymus grafts reconstruct a diverse and functional T‐cell repertoire while maintaining immune tolerance and minimizing pathological immune activation, highlighting the safety and therapeutic potential of the ISTR strategy.

### 2.4 T‐Cell Reconstitution of ISTR in Aged Mice and HSCT Mice

To further evaluate the performance of the ISTR system, we extended this strategy to aged mice and thymectomized recipients undergoing hematopoietic stem cell transplantation (HSCT).

**FIGURE 4:**
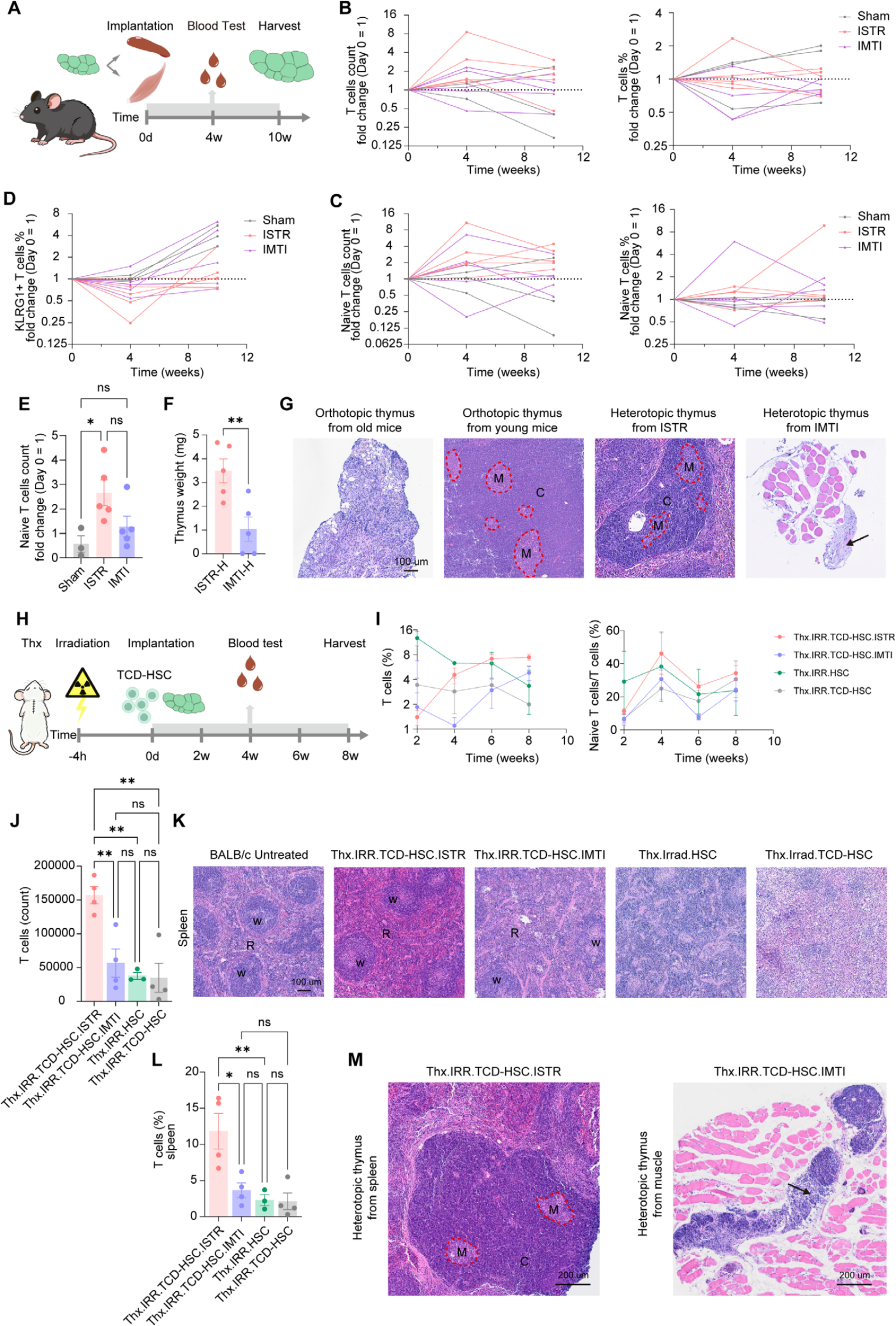
Reconstitution of T cells in aged mice and hematopoietic stem cell transplantation mice. (A) Experimental scheme: cultured thymic tissue was implanted in the spleen or quadriceps femoris of old mice. Blood was analyzed on day 0 before transplantation, at week 4, and at week 10 post‐transplantation. Thymus grafts from the spleen or quadriceps femoris, and orthotopic thymus from old mice were collected at week 10 post‐graft. (B–D) Changes in T‐cell counts and percentages in CD45^+^ lymphocytes (B), naive T‐cell counts and percentages in total T cells (C), and the percentage of KLRG1^+^ T cells in total T cells (D) at week 4 and week 10. Results were shown as fold changes, normalized to day 0 (Sham: n = 3, ISTR and IMTI: n = 5). (E) Fold change of naive T‐cell counts in old mice at 10 weeks post‐graft. (F) Weight of the heterotopic thymus from the spleen or quadriceps femoris at 10 weeks post‐graft. (G) Representative morphology of the orthotopic thymus or heterotopic thymus from the spleen or quadriceps femoris at 10 weeks post‐graft. Scale bar: 100 µm. (H) Experimental scheme of the intrasplenic thymus transplantation in HSCT: complete thymectomy was performed in BALB/c mice 7 days prior to X‐ray lethal irradiation; HSC or T‐cell‐depleted HSC from the bone marrow of C57BL/6J‐GFP mice were adoptively transferred to irradiated mice; cultured thymus was simultaneously transplanted into the spleen or quadriceps femoris of mice with T‐cell‐depleted HSC infusion. (I) Percentage of T cells or naive T cells in PBL from 2 to 10 weeks post‐graft (Thx.IRR.HSC group: n = 3, other cohorts n = 4). (J) Count of T cells in PBL at week 10 post‐graft. (K) Representative morphology of the spleen across different groups. Scale bar: 100 µm. (L) Percentage of T cells in the spleen at 10 weeks post‐graft. (M) Representative H&E staining images of heterotopic regenerated thymus tissue from the spleen or muscle. Scale bar: 200 µm. Data are mean ± SEM. Statistical significance was determined by one‐way ANOVA [(E) and (F), (I), and (K)]; ^*^ *p* < 0.05, ^****^ *p* < 0.0001; n.s., not significant.

In the aged mouse experiment, 20‐month‐old C57BL/6J mice were randomly assigned to ISTR, IMTI, and Sham groups (Figure 4A). Peripheral total T cells, KLRG1^+^ senescent T cells, and naive T cells were assessed on day 0, week 4, and week 10. Considering substantial intrinsic variability among aged individuals, data were normalized to day 0 baseline values for each animal. Although fold changes in T‐cell proportions fluctuated within each group—likely attributable to shifts in other leukocyte subsets, the ISTR group exhibited steeper upward trajectories in fold changes of total and naive T‐cell counts relative to the other two cohorts (Figure 4B,C). It is worth noting that the frequency of KLRG1^+^ senescent T cells was markedly reduced in the ISTR group at 4 weeks post‐graft and remained lower at 10 weeks compared with the other cohorts, indicating that the de novo‐generated cells modulated the percentage of senescent cell subsets (Figure 4D). The mean value of the fold change of blood naïve T‐cell counts in the ISTR group was higher than in the IMTI group at 10 weeks post‐transplantation, despite the absence of statistically meaningful differences. Additionally, the intrasplenic grafts outperformed the intramuscular grafts in terms of graft weight, histological structure and thymocyte cellularity (Figure 4F,G and Figure S16).

We next modeled the clinical challenge of insufficient T‐cell reconstitution post‐allogeneic HSCT using thymectomized BALB/c mice subjected to lethal irradiation. Mice were divided into four groups (Figure 4H and Figure S17A,B): Thx.IRR.TCD‐HSC.ISTR (T‐cell‐depleted HSC + ISTR), Thx.IRR.TCD‐HSC.IMTI (T‐cell‐depleted HSC + IMTI), Thx.IRR.TCD‐HSC (T‐cell‐depleted HSC only), and Thx.IRR.HSC (unmanipulated HSC only). We tracked peripheral T‐lymphocyte reconstitution by flow cytometry at biweekly intervals for up to 8 weeks post‐transplantation. The proportion of T cells progressively increased over time in the Thx.IRR.TCD‐HSC.ISTR group from week 2 post‐transplantation, while this trend began at a delayed time point of 4 weeks in the Thx.IRR.TCD‐HSC.IMTI group alongside a lower elevation magnitude; Thx.IRR.TCD‐HSC animals showed minor fluctuations without T‐cell repopulation, whereas Thx.IRR.HSC recipients exhibited continuous T‐cell loss (Figure 4I, left). Naive T‐cell frequencies transiently increased from week 2 to 4 across all groups, followed by mild oscillations, with the mean value of Thx.IRR.TCD‐HSC.ISTR group retaining the highest from week 4 until the endpoint (Figure 4I, right). Consistently, the peripheral T‐cell count was significantly higher in Thx.IRR.TCD‐HSC.ISTR mice than other groups at week 8 (Figure 4J), revealing robust rescue of severe T‐lymphopoietic insufficiency. As a consequence of lethal irradiation, spleen histology revealed disrupted architecture, with indistinct parenchymal structures and blurred boundaries between red and white pulp in Thx.IRR.TCD‐HSC mice (Figure 4K). Splenic tissue restoration was more complete in Thx.IRR.TCD‐HSC.ISTR recipients, whereas Thx.IRR.TCD‐HSC.IMTI displayed only mild histological improvement (Figure 4K). Unmanipulated HSC transfer in Thx.IRR.HSC mice triggered severe immune dysregulation, characterized by chaotic splenic architecture and diffuse lymphocyte infiltration (Figure 4K), consistent with life‐threatening graft‐vs.‐host disease (GVHD) that caused one animal death in this group. In line with peripheral blood data, Thx.IRR.TCD‐HSC.ISTR mice maintained the highest splenic T‐cell frequency (Figure 4L). Histological staining of regenerated grafts further validated these outcomes: ISTR grafts displayed superior thymic architecture, with a clearer, well‐populated cortico‐medullary structure (Figure 4M).

All the results above demonstrate the superior regenerative potency of ISTR under thymic involution or thymic ablation‐induced lymphopenic conditions.

### 2.5 Restoration of Antiviral and Antitumor Immunity Following T‐Cell Reconstitution

**FIGURE 5:**
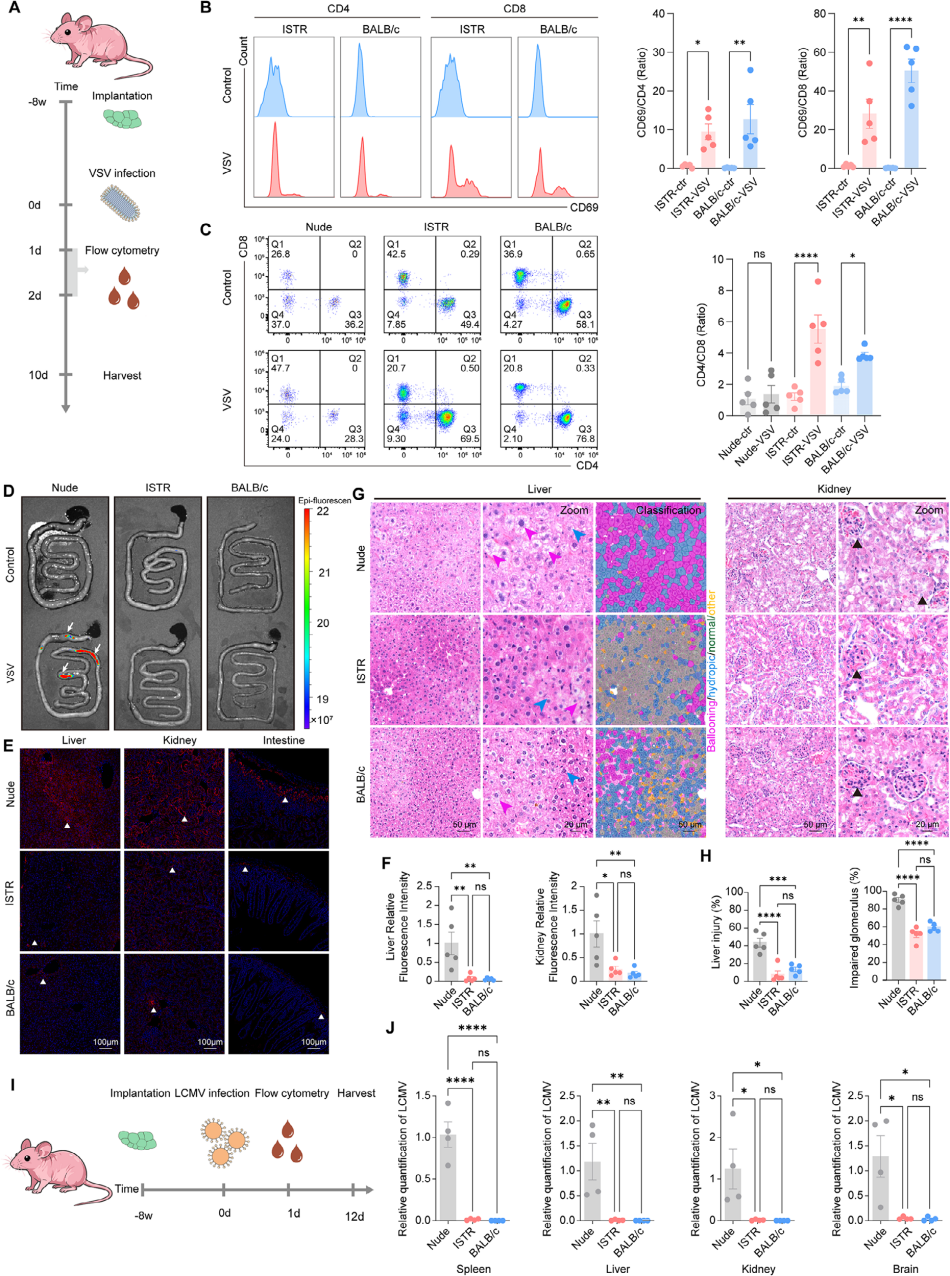
Intrasplenic thymic grafts reestablish protective immune responses. (A) Experimental outline: 12‐week‐old ISTR (8 weeks post‐graft), nude, and BALB/c mice received intraperitoneal VSV (5 × 10^6^ PFU) or PBS. Hematology and pathology were assessed at 24 h, 48 h, and day 10 post‐infection. (B) Up‐regulation of the activation marker CD69 on peripheral CD4+ and CD8^+^ T cells 24 h post‐VSV challenge (n = 5). (C) CD4:CD8 T‐cell ratio in blood at 48 h post‐infection (n = 5). (D) In vitro GFP imaging of the small intestine 10 days after VSV infection. (E to F) Representative immunofluorescence staining of liver, kidney, and intestine (E) and relative fluorescence intensity of VSV‐G protein (F) in sections of liver and kidney at 10 days post‐infection. Scale bar: 100 µm. (G) Representative H&E‐stained liver with hepatocytes classification masking and kidney sections at day 10 post‐infection. Blue arrows: hepatocyte hydropic change with mild cellular swelling and pale cytoplasm; red‐violet arrows: ballooning degeneration of the hepatocytes with cytoplasmic vacuolization or karyorrhexis; black triangles: glomerular congestion, hypercellularity, and obscuration of the capillary lumina. Scale bar: 50 µm, 20 µm. (H) Quantification of hepatic and renal injury scores from seven random fields per sample (n = 5). (I) Experimental outline: 12‐week‐old ISTR (8 weeks post‐graft), nude, and BALB/c mice received intraperitoneal LCMV Armstrong (3 × 10^5^ PFU) or PBS. Hematology and pathology were assessed at 24 h and day 12 post‐infection, respectively. (J) Relative expression of LCMV G protein in the spleen, liver, kidney, and brain across experimental groups (n = 4). Data are mean ± SEM. Statistical significance was determined by one‐way ANOVA [(B) and (C), (F), (H), and (J)]; ^*^ *p* < 0.05, ^****^ *p* < 0.0001; n.s., not significant. All experiments were independently repeated twice.

To evaluate the functional competence of the regenerated immune system, we first examined the immune response to viral infection. Nude mice, ISTR mice, and BALB/c mice received intraperitoneal injections of GFP‐labeled Vesicular Stomatitis Virus (VSV, 5 × 10^6^ PFU), with control groups receiving equivalent volumes of phosphate‐buffered saline (PBS) (Figure 5A). Peripheral blood was analyzed by flow cytometry at 24 and 48 h post‐infection. Mice were sacrificed 10 days post‐infection for sample collection. At 24 h post‐infection, CD69 expression—the earliest lymphocyte activation marker—significantly increased in peripheral blood CD4+ and CD8+ T cells in ISTR and BALB/c mice (Figure 5B), indicating that the splenic thymus‐regenerated T‐cell population mounted a normal antiviral response. Crucially, by 48 h post‐VSV infection, the CD4^+^/CD8^+^ T‐cell ratio in ISTR mice, as in BALB/c mice, increased significantly, indicating normal CD4^+^ T‐cell activation and proliferation upon viral challenge. In contrast, the nude mouse group showed no significant change in ratio (Figure 5C). Consistent with VSV pathogenesis timelines, mice were sacrificed on day 10. The persistently elevated spleen index in VSV‐infected nude mice indicated ongoing high‐level viral activity. In contrast, indices in the ISTR and BALB/c groups returned to pre‐infection levels (Figure S18A,B), signifying effective viral control and clearance. GFP fluorescence imaging of intestinal tissue confirmed substantial residual virus in nude mice, whereas near‐complete clearance was observed in ISTR and BALB/c mice (Figure 5D). Immunofluorescence staining for VSV‐G protein further delineated viral load differences: nude mouse livers and kidneys exhibited higher intensity of VSV‐G^+^ signals, while ISTR and BALB/c tissues showed smaller positive areas and lower intensities (Figure 5E,F). Strong VSV‐G signals permeated the nude mouse intestinal wall, contrasting with minimal scattered signals in the intestines of ISTR and BALB/c mice, demonstrating that splenic thymus transplantation effectively contained viral replication and dissemination (Figure 5E). Histopathological assessment revealed severe VSV‐induced damage in nude mice. VSV‐induced loss of intestinal mucosal integrity and lamina propria inflammatory infiltration were alleviated in ISTR and BALB/c mice (Figure S18C). Liver sections displayed characteristic hepatocyte degeneration, including extensive hydropic degeneration (reticulated, vacuolated cytoplasm) and scattered ballooning degeneration (rarefied cytoplasm, nuclear pyknosis, karyorrhexis)‐lesions markedly reduced in ISTR and BALB/c groups (Figure 5G,H). Kidney pathology was consistent with liver findings: ISTR and BALB/c mice exhibited significantly less glomerular congestion, hypercellularity, and boundary blurring than nude mice (Figure 5G,H). These data demonstrated that the regenerated T‐cell compartment mounts effective antiviral immunity, controlling viral replication and preventing tissue damage.

In addition, we utilized the well‐established Armstrong strain of lymphocytic choriomeningitis virus (LCMV), a classic viral model for evaluating T cell function, to further verify the antiviral capacity of the ISTR system. Nude, ISTR, and BALB/c mice were challenged via intraperitoneal injection with 3 × 10^5^ PFU LCMV, while control animals received an equal volume of PBS. Peripheral blood was collected at 24 h post‐infection, and solid organs, including the spleen, liver, kidney, brain, and lymph nodes, were harvested on day 12 post‐infection (Figure 5I). Relative quantification of LCMV glycoprotein transcripts in the spleen, liver, kidney, and brain on day 12 revealed drastically reduced viral loads in LCMV‐infected ISTR and BALB/c mice relative to nude controls (Figure 5J), indicative of robust viral suppression and clearance. Concordant results were observed in LCMV immunofluorescence staining of lymph nodes, which exhibited fewer LCMV‐positive signals in ISTR and BALB/c mice compared with nude animals (Figure S18D). Collectively, these findings, combined with our VSV infection data, firmly establish that ISTR mice mount potent antiviral responses and possess prominent antiviral protective capacity.

Having confirmed antiviral competence, we next evaluated antitumor immunity using murine tumor challenge and xenograft models. Murine B16 melanoma cells were transplanted subcutaneously into the flanks of nude, ISTR, and BALB/c mice. Tumors were excised and weighed after 18 days (Figure S19A). B16 tumor volume in ISTR mice was significantly smaller than in nude mice and comparable to BALB/c controls (Figure S19B). H&E and immunofluorescence staining of tumor sections revealed robust infiltration of immune cells (H&E), T cells (CD3^+^), and CD8^+^ T cells at tumor margins and interiors in ISTR mice—infiltration largely absent in nude mice (Figure S19C), indicating that regenerated T cells correctly recognized and restricted tumor growth. Finally, we tested the tumorigenicity of human tumor xenografts. Human HCT116 colon carcinoma cells—which form tumors in 100% of nude mice—were transplanted. ISTR mice, benefiting from reconstituted T‐cell immunity, exhibited 0% tumor formation, matching that of immunocompetent mice and confirming the complete restoration of xenotransplant rejection capability and comprehensive immune surveillance (Figure S19D–F). These results demonstrate that the regenerated T cells not only mount specific antitumor responses but also infiltrate tumors and function within them.

Together, these findings across viral, tumor, and xenograft models demonstrate that ISTR restores multifunctional T‐cell immunity capable of mounting appropriate responses to diverse immune challenges, with efficacy matching that of intact immunocompetent systems.

### 2.6 Assessment of Graft Resilience and Durability

Having established that the ISTR system confers superior thymic function and naive T‐cell output, we next evaluated the stress resilience of the regenerated grafts via two complementary assays: sublethal irradiation challenge and secondary serial transplantation.

To profile the dynamic recovery of thymocytes following irradiation, ISTR and IMTI mice at 6 weeks post‐transplantation, along with age‐matched BALB/c mice, received 3 Gy total‐body X‐ray irradiation, namely ISTR.IRR, IMTI.IRR and BALB/c.IRR groups. Graft weight and total cellularity were determined on day 7, day 14, and day 35 post‐irradiation (Figure S20A). Graft weight followed a similar temporal trajectory in both groups: mass was low at day 7, rebounded by day 14, then declined again at day 35, indicative of an early transient recovery phase. Thymic cellularity surged at day 14 in ISTR.IRR, while no signs of recovery were observed in IMTI.IRR. Notably, graft weight and cellularity were substantially higher in ISTR.IRR recipients relative to IMTI.IRR mice at days 14 and 35, demonstrating enhanced regenerative capacity of ISTR grafts (Figure S20B,C).

During the early recovery phase (days 7–14), ISTR.IRR grafts exhibited elevated double‐positive (DP) populations, then declined again at day 35, uniformly within IMTI.IRR grafts (Figure S20D). DN3 progenitors, one of the most vulnerable populations to radiation, progressively diminished in IMTI.IRR grafts over time, pointing to severe developmental arrest at the DN stage (Figure S20E). Circulating naive T‐cell counts dropped in both ISTR.IRR and IMTI.IRR mice at day 7 post‐irradiation; however, ISTR.IRR mice displayed a clear recovery trend by day 35, while naive T cells remained persistently depleted in IMTI.IRR animals. Consistently, naive T‐cell frequency markedly declined in IMTI.IRR grafts but remained largely preserved in the ISTR.IRR group (Figure S20F), implying sustained de novo T‐cell replenishment supported by the ISTR.IRR graft. Histology of ISTR.IRR grafts showed reduced thymocytes on day 7 and gradual recovery over time, while recovery of IMTI.IRR grafts showed repopulation on day 14 but marked involution on day 35 (Figure S20G), further confirming the irradiation‐induced degeneration. These data indicate that ISTR grafts possess superior resilience against sublethal radiation.

We further tested the durability of the grafts using secondary transplantation. Primary thymic grafts were harvested at 6 weeks post‐transplantation and reimplanted into the second batch of 4‐week‐old nude recipients at the same site as the primary transplantation (Figure S21A), with age‐matched untransplanted nude mice serving as controls. Peripheral blood, splenic T‐cell compartments, and secondary thymic grafts were analyzed at 6 weeks after serial transplantation. Results showed that splenic T‐cell proportion was significantly elevated in recipients of serially transplanted ISTR grafts (ISTR‐ISTR) compared to the IMTI‐IMTI group; blood T‐cell ratio in the ISTR‐ISTR group was also higher than in the IMTI‐IMTI group, but showed no significant differences due to substantial intra‐group variation (Figure S21B), implying the enhanced T‐cell output in the ISTR‐ISTR group. In parallel, secondary ISTR grafts exhibited markedly larger tissue diameters than secondary IMTI grafts (Figure S21C), with one IMTI‐derived graft showing near‐complete atrophy at harvest. Combined with the irradiation challenge data, these results verify that ISTR‐derived thymic grafts exhibit superior tissue stability and stress resistance upon repeated serial transplantation.

### 2.7 Enhancement of Human T‐Cell Repopulation via ISTR

**FIGURE 6:**
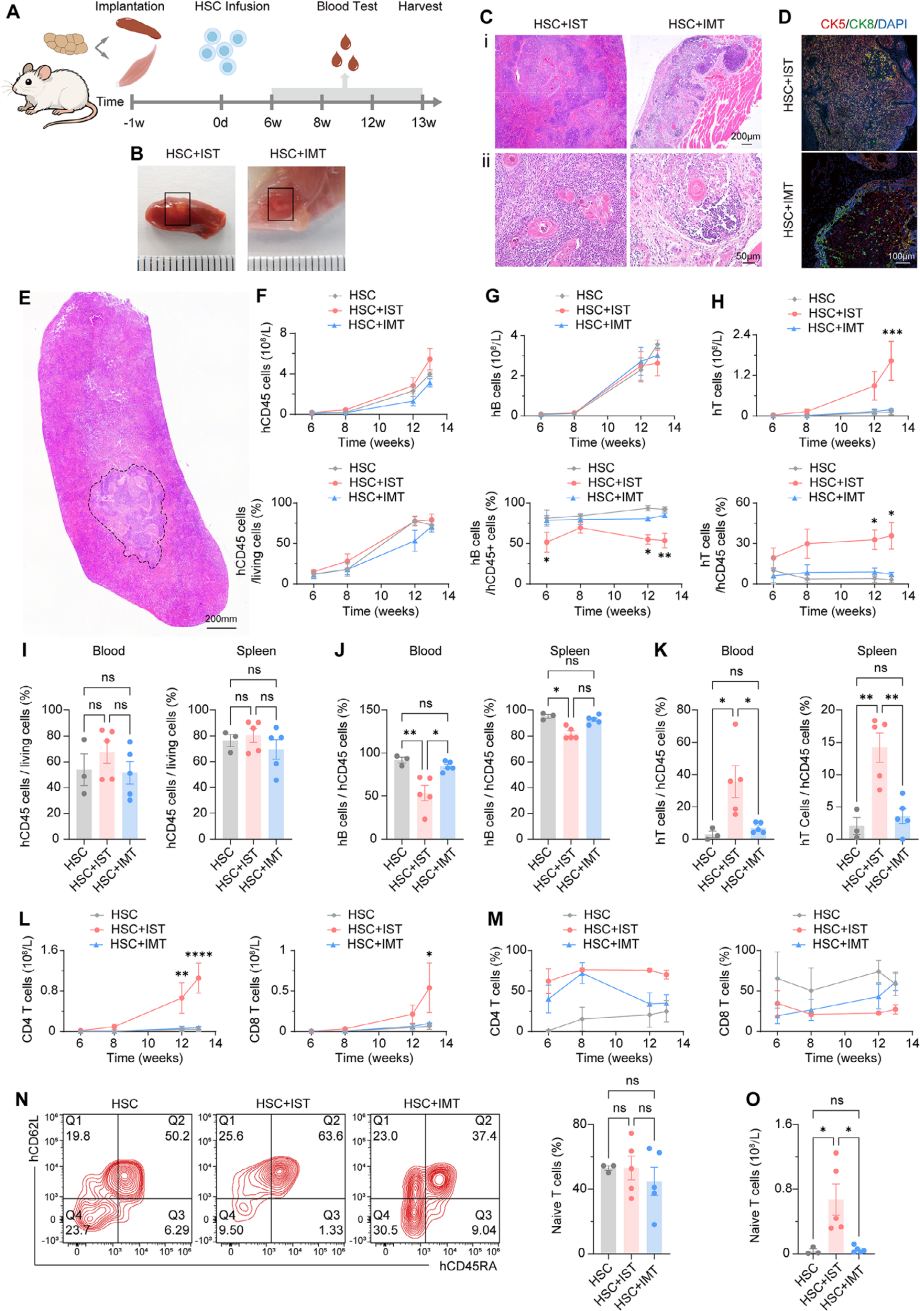
Intrasplenic thymus transplantation augments human T‐cell reconstitution in HSC‐humanized mice. (A) Experimental timeline: human thymic fragments were transplanted into the spleen or muscle of NCG‐X mice one week before intravenous infusion of human CD34^+^ HSCs; analyses were performed up to week 13. (B) Gross morphology of intrasplenic (HSC + IST) and intramuscular (HSC + IMT) thymic grafts at week 13. (C) (i) H&E staining of human thymic tissue within spleen and muscle at week 13. (ii) Higher‐magnification view of Hassall's corpuscles in grafts. Scale bars: 200 µm (i), 50 µm (ii). (D) Immunofluorescence for CK8 (cortex) and CK5 (medulla) in splenic vs. muscular grafts at week 13. Scale bar: 100 µm. (E) Macroscopic image of a thriving intrasplenic thymic graft (dotted outline). Scale bar: 200 mm. (F) Absolute counts and percentages of human CD45^+^ leukocytes in peripheral blood (PB) post‐HSC infusion (n = 3–5). (G, H) Absolute numbers and proportions of human CD19^+^ B cells (G) and CD3^+^ T cells (H) in PB across HSC‐only, HSC + IST, and HSC + IMT groups. (I–K) Frequencies of CD45^+^ leukocytes (I), B cells (J), and T cells (K) in the PB and spleen at week 13. (L, M) Absolute counts (L) and percentages (M) of CD4^+^ and CD8^+^ T‐cell subsets in CD3^+^ T cells. (N, O) Flow‐cytometric analysis of naïve T cells (hCD45RA^+^hCD62L^+^): representative plots (N, left), frequencies (N, right), and absolute counts (O) in each group. Data are mean ± SEM (n = 3 for HSC‐only; n = 5 for HSC + IST and HSC + IMT). Statistical tests: one‐way ANOVA [(I) to (K), (N) and (O)] and two‐way ANOVA [(F) to (H), (L) and (M)]; ^*^ *p* < 0.05, ^****^ *p* < 0.0001; n.s., not significant. Panels B–E were repeated in three independent experiments.

To assess the translational potential of intrasplenic thymic transplantation, we used cryopreserved thymic tissue and hematopoietic stem cells (HSCs) from the same pediatric donor to generate humanized mice. NCG‐X mice, which lack functional NK, T, B, and myeloid cells, were used as recipients. Mice were divided into five groups (Figure 6A): control (NCG‐X), intrasplenic thymus‐only (IST), HSC‐only (HSC), HSC + intrasplenic thymus (HSC + IST), and HSC + intramuscular thymus (HSC + IMT). Thymic tissue was transplanted at 5 weeks of age, followed by the intravenous injection of 1 × 10^5^ HSCs one week later. Peripheral blood was monitored weekly for human CD45^+^, CD3^+^, CD19^+^, CD4^+^, and CD8^+^ cells starting at week 6 post‐HSC transplantation. Mice were then sacrificed at week 13 for analysis. At week 13, intrasplenic thymic grafts were visibly larger than muscle grafts (Figure 6B). Histologically, intrasplenic grafts showed intact epithelial architecture with no necrosis. In contrast, intramuscular grafts frequently displayed necrotic regions and limited intact thymic structure (Figure 6C,D). In some cases, intramuscular grafts became undetectable, possibly due to tissue degradation. Panoramic H&E staining of HSC + IST spleens revealed extensive cortico‐medullary organization, indicating robust growth of human thymic tissue in the spleen (Figure 6E). These findings demonstrate that the splenic microenvironment provides optimal conditions for human thymic graft survival and architectural maintenance.

As thymocytes are sensitive to cryopreservation, few human T cells were detected in the peripheral blood of IST‐only mice within 12 weeks (Figure S22A). In contrast, HSC‐transplanted groups exhibited clear T‐cell reconstitution (Figure S22B,C), indicating that most human T cells were derived from HSCs that matured within the thymic graft. Accordingly, subsequent analyses focused on the HSC, HSC + IST, and HSC + IMT groups. Across the 13 weeks, no significant differences were observed in total human CD45^+^ cells or B‐cell counts among the three groups (Figure 6F,G, top and Figure S22D,E), suggesting comparable overall engraftment. However, the HSC + IST group had a lower proportion of B cells (Figure 6G, bottom) and significantly higher numbers and proportions of T cells (Figure 6H and Figure S22B). At week 13, despite similar levels of total human CD45^+^ cells in the blood and spleen (Figure 6I), the HSC + IST group exhibited a higher T‐cell ratio and lower B‐cell ratio (Figure 6J,K), consistent with accelerated or enhanced T‐cell output from splenic grafts. Further analysis showed that CD4^+^ and CD8^+^ T‐cell counts were significantly higher in the HSC + IST group than in the HSC + IMT group (Figure 6L). At week 6, CD4^+^ T cells had become the predominant T‐cell subset (> 50%) in HSC + IST mice, whereas CD8^+^ T cells predominated in the other groups (Figure 6M), consistent with findings in nude mice and suggesting site‐dependent differences in T‐cell subset output. These results establish that splenic thymic transplantation supports more robust and balanced T‐cell reconstitution than intramuscular implantation.

Crucially, the T cells generated through intrasplenic thymic transplantation demonstrated enhanced functional capacity. At week 13, the number of human naïve T cells (hCD62L^+^hCD45RA^+^) was significantly higher in the HSC + IST group, although their relative proportions were comparable across groups (Figure 6N,O and Figure S22F), indicating enhanced functional T‐cell output. After stimulation, CD4^+^ T cells from the HSC + IST group could differentiate into Th1, Th2, Th17, or Treg subsets (Figure S22G). Together, these data validate that intrasplenic thymic transplantation promotes quantitatively enhanced naïve T‐cell reconstitution and maintains CD4^+^ T‐cell differentiation capacity.

In summary, our humanized mouse model provides compelling evidence for the translational potential of intrasplenic thymic transplantation. The splenic microenvironment not only ensures superior graft survival and structural organization but also enables enhanced T‐cell reconstitution with balanced subset distribution and functional competence. These findings position intrasplenic transplantation as a potential preclinical model and promising clinical strategy for thymic regeneration therapy.

## 3 Discussion

Restoring thymic function represents an urgent unmet need in both clinical and experimental immunology. In this study, we have devised an innovative ISTR technique that enables rapid, complete reconstitution of T‐cell‐mediated immunity in athymic mice. Robust validation of this approach is provided by the restoration of a highly diverse T‐cell repertoire, augmented immune responses in nude mice, and rapid functional reconstitution of T cells in thymectomized HSCT mice and humanized mice engrafted with human thymic tissue and HSCs. Unlike previously reported thymic transplantation methods, ISTR achieves complete thymic organogenesis from injectable tissue fragments. The regenerated thymic grafts establish a structurally intact, autonomous organ within the splenic parenchyma that is functionally equivalent to the native thymus in its capacity to generate a de novo T‐cell compartment.

Although the ISTR group displayed clearer T‐cell recovery and stronger tissue regeneration than IMTI in aged mice, intergroup fold changes in naive T‐cell numbers lacked statistical significance. One of the vital contributors involves the aged bone marrow hematopoietic progenitors, which exhibit defective lymphoid priming and thus constrain sustained thymopoiesis [45, 46]. Additionally, fibrotic aged secondary lymphoid niches lack sufficient IL‐7 to support recent thymic emigrants, driving premature T cell dysfunction [47]. Critically, persistent systemic pro‐inflammatory cytokines and resident senescent T cells compete for survival niches and impose exhausted phenotypes on graft‐derived new T cells [48]. Thus, despite boosting de novo T cell output, a thymus graft alone cannot comprehensively rejuvenate aged T‐cell immunity. However, in the future, when combined with young bone marrow transplantation and senolytic treatment, ISTR may facilitate more robust T‐cell recovery in aged mice.

The spleen's uniquely permissive microenvironment underlies ISTR's success, a feature we have corroborated in our recent studies on liver, thyroid, and islet regeneration [49, 50, 51]. This microenvironment confers four key advantages for thymic engraftment and function. First, the spleen's sinusoidal vasculature intimately perfuses engrafted fragments with circulating blood even prior to neovascularization, thereby preventing ischemia‐reperfusion injury and allowing time for graft accommodation. Second, the spleen's loosely organized stromal network imposes minimal external pressure on implanted tissues, in contrast to the compressive milieu encountered in muscular or subcutaneous sites, thus preserving nutrient and oxygen diffusion. Third, the high plasticity of splenic vessels facilitates rapid angiogenic remodeling, reestablishing a normal blood supply within a short timeframe. Fourth, resident macrophages and other immune cells within the spleen may contribute to general regenerative signaling and thymus‐specific niche cues, a hypothesis requiring comparative analysis across multiple organ‐spleen transplantation models. Additionally, as a potential hematopoietic organ under several conditions [37], the spleen may also be a reservoir of hematopoietic precursor cells to further enhance thymopoiesis.

Although concerns may arise regarding the safety of intraparenchymal splenic injections, our nonhuman primate (NHP) studies have demonstrated the feasibility and favorable safety profile of this procedure [51]. Under ultrasound guidance, percutaneous splenic injection in macaques induced no detectable alterations in splenic consistency or hematological indices. Given the high anatomical and physiological homology between NHP and human spleens, these findings strongly support the translational relevance of ISTR for clinical implementation.

In humanized mouse studies, fetal thymic fragments engrafted within the spleens of immunodeficient hosts likewise demonstrated thymic survival and robust thymopoiesis, yielding functional human T‐cell populations. This dual demonstration—murine thymus regeneration and human thymopoiesis—underscores the universal importance of prompt vascular support for thymic graft survival and also suggests that ISTR may be effectively translated to human patients. The generation of mature human T cells further attests to the clinical promise of this approach.

Despite the inherent risk of GVHD in allogeneic thymus transplantation [22, 25], we observed only sporadic GVHD in ISTR mice, markedly lower than in IMTI mice. We attribute this favorable safety profile to three principal factors: (1) preservation of native thymic architecture within the splenic niche, which enhances negative selection of autoreactive thymocytes; (2) continuous delivery of self‐antigens by autologous apoptotic splenocytes and self‐antigen‐presenting cells, promoting central tolerance [52, 53]; and (3) abundant exposure of recent thymic emigrants to tolerogenic splenic APCs, reinforcing self‐tolerance. Observations in splenectomized transplant recipients—who exhibit increased graft rejection [54]—corroborate the spleen's pivotal role in systemic immune tolerance. Specialized splenic domains, including the marginal zone and T‐cell‐enriched areas, harbor stromal and dendritic cell subsets that avidly capture apoptotic debris [28, 55], as well as macrophage subsets that recognize apoptotic cells and secrete TGF‐β and IL‐10 [29] and drive regulatory T‐cell differentiation [31], thereby attenuating autoimmunity. The precise mechanisms warrant further systematic investigation.

The primary clinical indication for ISTR is congenital thymic hypoplasia or athymia, for which existing intramuscular thymus implants restore T‐cell immunity only slowly and incompletely [26]. Our data demonstrate that ISTR offers a more robust and comprehensive reconstitution of thymic function and the T‐cell compartment. Beyond the rising diagnoses of congenital athymia [56, 57], there exists a pressing clinical need to restore immunity after severe insults (e.g., severe infection, T‐cell ablation, or myeloablative conditioning), especially when accompanied by irreversible thymic impairment. This need, coupled with the ongoing demand to induce tolerance in transplantation, makes the pursuit of rapid, effective, and safe thymic regeneration strategies a paramount therapeutic goal. Moreover, preclinical evidence suggests that regenerated thymus may ameliorate autoimmune disorders [58, 59] and age‐related immunosenescence [60, 61]. ISTR therefore provides a versatile, efficient platform for both therapeutic and investigative applications.

Beyond its clinical promise, ISTR represents a powerful tool for immunological research. Its minimally invasive approach allows implantation of larger thymic fragments compared to subcapsular or intramuscular methods, and, critically, its supportive niche better fosters the growth and maturation of the transplanted thymic tissue as well as its resident thymocytes, enabling the generation of fully reconstituted T‐cell repertoires without compromising adjacent organs. When combined with emerging synthetic‐biology techniques—such as engineered thymic stromal networks or 3D‐organoid co‐culture systems—ISTR may facilitate more sophisticated studies of thymic architecture, central tolerance, and T‐cell repertoire diversity. In summary, ISTR accelerates thymic tissue regeneration in both nude and humanized mouse models, markedly reduces the risk of GVHD, and establishes a robust foundation for future advances in immune reconstitution strategies.

## 4 Conclusion

In this study, we established the ISTR strategy and characterized its potency in thymic growth and T‐cell output in thymic hypoplasia, thymic involution, and thymectomized mouse models, as well as in a humanized mouse model. Our results demonstrate that ISTR surpasses IMTI in supporting tissue development and generating diverse T‐cell populations under multiple experimental conditions. This capacity is critical for maintaining intact immune function in the face of thymic dysfunction. These findings highlight the potential of ISTR in thymic regeneration and T‐cell reconstitution and motivate further exploration of approaches to regulate the production of diverse T‐cell populations and central tolerance.

## 5 Methods

### 5.1 Experimental Design

The overall objective of this study was to evaluate the effect and advantages of thymus regeneration in the spleen. Thymus tissue from C57BL/6J EGFP^+^ mice was cultured and transplanted into the spleens or muscles of BALB/c nude mice, aged mice, or thymectomized mice. Human thymic tissue was grafted into NCG‐X mice, followed by human HSC transplantation. Regenerated thymic tissue, T‐cell output, and function were monitored via histological, hematological, and serum analyses. All mice were female to minimize gender‐related variability and androgen‐related effects. Mice were randomly assigned to control and experimental groups, and the study was not blinded. The number of independent experiments (n) is indicated in the figure legends. Data collection was stopped at the predetermined times. No data were excluded from the analysis. Statistical analysis was performed by two‐tailed *t*‐test (two groups) and one‐way or two‐way analysis of variance (ANOVA) followed by Tukey's multiple comparisons test (multiple groups).

### 5.2 Materials and Methods

#### 5.2.1 Animals

NCG‐X mice (NOD/ShiLtJGpt‐Prkdc*^em26Cd52^* Il2rg*^em26Cd22^* kit*^em1Cin(V831M)^*/Gpt, strain NO. T003802), BALB/c mice, BALB/c nude mice, and C57BL/6J EGFP^+^ mice (C57BL/6JGptH11*^em1Cin (CAG‐EGFP‐polyA)^*/Gpt) at 4–8 weeks of age were purchased from Gempharmatech (Jiangsu, China). The mice were maintained in a specific pathogen‐free animal facility with controlled temperature, humidity, and a 12‐h light–dark cycle. All animal procedures were under ethical approval by the Institutional Animal Care and Use Committee of Nanjing University (approval no. IACUC‐2111005‐2) and the Model Animal Research Center of Nanjing University (AP#: LY‐01, LY‐02), and conformed to the Guide for the Care and Use of Laboratory Animals published by the National Institutes of Health. Human CD34^+^ cells described in this article were obtained from fetal liver samples under clinical ethical approval from the ethical committee of Drum Tower Hospital with informed consent (protocol#: 2021‐488‐01 for FL).

#### 5.2.2 Thymus Processing and Culture

Fresh thymus was isolated from neonatal C57BL/6J EGFP^+^ mice (0–2 days old). For fresh thymus implantation, the thymus was cut into small blocks of approximately 0.2, 0.5, 0.7 and 0.9 mm in diameter, respectively, and then rinsed with sterile PBS before gentle shaking in RPMI 1640 medium (Gibco, 72400047) with Fetal Bovine Serum (FBS, Gibco, A5669701) at 37°C for 5 min on the shaking table.

For cultured tissue transplantation, the thymus was cultured for a week as previously reported [62] with slight modifications. Briefly, the thymus lobes were longitudinally cut with a small incision, followed by gentle shaking in 1640/10% FBS medium at 37°C for 5 min. The thymic lobes were then placed on gelatin sponges soaked in DMEM/F12 (Gibco, C11330500BT) supplemented with 10% FBS and 2'‐deoxyguanosine (1.35 mm, Cayman, 9002864), and incubated at 37°C, 5% CO_2_ for a week with daily medium replacement. The cultured tissue was examined by H&E staining and flow cytometric analysis one day before implantation. Tissues were weighed and trimmed to a uniform weight of 0.5–0.6 mg prior to transplantation. Samples were divided into about 12, 8, 4, and 2 fragments for the 0.2, 0.5, 0.7, and 0.9 mm groups, respectively, with minimal tissue continuity retained between pieces.

Human thymus and HSCs were obtained with ethical approval from the Medical Ethics Committee of Affiliated Drum Tower Hospital, Medical School of Nanjing University. The processing of the cryopreserved human thymus tissue after thawing was similar to that of the fresh mouse thymic tissue.

#### 5.2.3 Transplantation of Thymus Tissue

The thymic tissue was washed in cold PBS. After the mouse was anesthetized, the tissue suspension was injected into the host spleen using an intravenous infusion needle. A gelatin sponge was used to control bleeding. Alternatively, the lateral thigh muscles of mice were used as a comparison site for thymus transplantation. Small incisions were made along the muscle fibers. The cultured thymic tissue was then processed and buried, followed by suture.

#### 5.2.4 Thymic Graft Isolation

Thymic tissue was exposed under the GFP excitation laser, and images were captured with the corresponding filter. Tissue was isolated with the assistance of glasses with a GFP filter, and trimmed, then washed 3–4 times to remove excess non‐thymic tissue.

#### 5.2.5 Thymus Transplantation in Aged Mice

Blood from 20‐month‐old female C57BL/6J mice was collected on day 0 before thymus transplantation. Mice were randomly assigned to the Sham, ISTR, and IMTI groups and received a sham operation, intrasplenic or intramuscular thymic transplantation with cultured C57BL/6J‐GFP mice‐derived neonatal thymi. Blood was evaluated at the fourth and 10th week post‐transplantation. Thymic tissue was collected at the 10th week post‐graft, weighed, and analyzed by flow cytometry.

#### 5.2.6 Adult Mouse Thymectomy

Mice (female, 6‐week‐old) were anesthetized via intraperitoneal injection of tribromoethanol (250 mg/kg). Chest fur was shaved, and the surgical field was disinfected with 70% ethanol and iodophor under aseptic conditions. A 0.8–1 cm longitudinal incision was made at the suprasternal notch, followed by blunt separation of subcutaneous fascia and pectoral muscles to expose the superior mediastinum. The sternum was slightly split at the manubrium with microscissors, and the bilateral thymic lobes were visualized under a dissecting microscope. Both thymic lobes were completely excised with fine forceps and microscissors, without damaging the heart or great vessels. After confirming full removal of thymic tissue, the sternum, muscle layers, and skin were sequentially sutured with absorbable polyglactin sutures. Postoperative analgesia was provided by subcutaneous injection of buprenorphine (0.05 mg/kg) every 12 h for two consecutive days. Mice were maintained on a thermostatically controlled heating pad until full recovery from anesthesia. Complete thymectomy was verified by post‐mortem mediastinal inspection at experimental endpoints.

#### 5.2.7 Total Body Irradiation

To establish a mouse model of irradiation‐induced injury, mice were subjected to a single dose of total body X‐ray irradiation. Before irradiation, mice were placed in well‐ventilated irradiation chambers to minimize movement during exposure and to ensure consistent dose delivery. Each mouse received 3 Gy (sublethal irradiation) or 8 Gy (lethal irradiation) of total body irradiation using an RS‐2000‐PRO‐225 X‐ray irradiator (Rad Source, USA) at a dose rate of 0.9 Gy/min. Unirradiated control mice were handled under the same conditions but were not exposed to X‐ray irradiation. After irradiation, mice were returned to the SPF animal facility and maintained under standard conditions until sacrifice at the indicated time points for subsequent analyses.

#### 5.2.8 Allogeneic Hematopoietic Stem Cell Transplantation

6‐week‐old female BALB/c mice were thymectomized and used as recipients of allo‐HSCT. Donor HSCs were isolated from the bone marrow of 6–8‐week‐old female C57BL/6J‐GFP mice. T‐cell‐depleted HSCs (TCD‐HSC) were purified from HSCs via negative magnetic bead separation. Intravenous HSC infusion (5 × 10^6^ per mouse) was performed 3–4 h after lethal irradiation of 8 Gy, following cultured thymic tissue implantation into the spleen or the quadriceps femoris muscle subsequently as previously described.

#### 5.2.9 Second Transplantation of the Thymic Graft

Equivalent weights of cultured thymic tissues were transplanted into the spleen or quadriceps femoris muscle of 4‐week‐old female nude mice. Thymic grafts were isolated at 6 weeks after the primary transplantation. The primary grafts were washed, minced as previously described, and introduced into the same site as the primary transplantation (intrasplenic graft to spleen, intramuscular graft to quadriceps femoris muscle) of the second batch of 4‐week‐old nude mice. Blood and spleen were collected and analyzed at week 6 post the second transplantation.

#### 5.2.10 Mouse Immunization and Spleen Collection

BALB/c and ISTR mice were immunized by intraperitoneal injection on day 0 with 100 µg ovalbumin (OVA) protein (MedChemExpress, Cat. No. HY‐W250978), 25 µg poly (I:C) (MedChemExpress, Cat. No. HY‐107202), 25 µg R848 (TargetMol, Cat. No. T6964_1–10 mg), and 25 µg CpG (MedChemExpress, Cat. No. HY‐150217) in a total volume of 200 µL sterile PBS per mouse. Mice received a booster immunization on day 14 with the same formulation, dose, injection volume, and route. Seven days after the final immunization, mice were euthanized, and spleens were harvested under sterile conditions for subsequent immune cell isolation and functional assays.

#### 5.2.11 In Vitro T‐Cell–Dendritic Cell Coculture Assay

Spleens were collected from immunized mice and mechanically dissociated to generate single‐cell suspensions. After red blood cell lysis and washing, T cells were purified from splenocytes by negative selection using a mouse T‐cell isolation kit according to the manufacturer's protocol (eynoslifescience, Cat. No. SN4101L).

Dendritic cells were isolated from splenic immune cells of BALB/c and C57BL/6 mice, respectively. CD11c^+^ dendritic cells were enriched by positive magnetic selection using CD11c MicroBeads following the manufacturer's instructions (Miltenyi Biotec, Cat. No. 130‐125‐835).

For antigen loading and activation, 1 × 10^5^ CD11c^+^ dendritic cells were incubated overnight with OVA protein (20 µg/mL), followed by LPS (750 ng/mL) stimulation for 6 h in complete RPMI 1640 medium at 37°C in a humidified 5% CO_2_ incubator. Complete RPMI 1640 medium was composed of RPMI 1640 supplemented with 10% FBS, 1 mm sodium pyruvate, 1× nonessential amino acids, 10 mm HEPES, and 50 µm 2‐mercaptoethanol. After incubation, dendritic cells were washed thoroughly to remove unbound OVA and residual LPS.

The antigen‐loaded dendritic cells were co‐cultured with 1 × 10^6^ purified T cells in complete RPMI 1640 medium at 37°C with 5% CO_2_. After 18 h, BFA was added, and cells were incubated for an additional 4 h. Cells were then harvested and processed for flow cytometric staining and analysis.

#### 5.2.12 Generation of Humanized Mice

Generation of humanized mice was described as previously reported [63, 64]. In brief, fetal liver CD34^+^ cells were enriched by affinity chromatography and assessed for purity. Six‐week‐old NCG‐X mice were injected via the tail vein with 1 × 10^5^ human FL CD34^+^CD38^−^ cells in 200 µL RPMI 1640 (Biological Industries, Cat# 01–100‐1ACS) with a 29‐gauge needle (BD Biosciences, Cat# 328421). Human immune cell reconstitution in humanized mice was monitored at 6, 8, 12, and 13 weeks via flow cytometry of peripheral blood.

#### 5.2.13 VSV Infection

VSV‐GFP strain, containing a GFP coding gene, was derived from the wild‐type Indiana strain and exhibited similar replication kinetics to the wild‐type Indiana strain [65], which was generously provided by Dr. Deyan Chen of the Medical School of Nanjing University, and initially established by JK Rose's lab at Yale University [66]. Nude, ISTR, and BALB/c mice (12 weeks old, female) were randomly divided into two groups, including the control group (n = 6) and the VSV infection group (n = 6). Mice of the infection group were infected with VSV (5 × 10^6^ PFU) or with DMEM by intraperitoneal injection on day 0. Samples were then collected and analyzed on days 1 and 2. Subsequently, the mice were euthanized on day 10, and their organs and blood were collected for further detection.

#### 5.2.14 LCMV Infection

LCMV Armstrong strain was kindly provided by Dr. Lilin Ye at Third Military Medical University, and originally obtained from Dr. Rafi Ahmed at Emory Vaccine Center. Nude, ISTR, and BALB/c mice (12 weeks old, female) were randomly divided into two groups, including the PBS group (n = 4) and the LCMV infection group (n = 4). Mice of the infection group were infected with LCMV (3 × 10^5^ PFU) or with PBS by intraperitoneal injection on day 0. Mice were euthanized on day 12, and samples were collected for further detection.

#### 5.2.15 Immune Repertoire

TCR β‐chain (TRB) repertoire was profiled from RNA samples using the ImmuHub TCR profiling system (ImmuQuad Biotech, China). Briefly, a 5′ RACE unbiased amplification protocol was used to control bottlenecks and eliminate PCR and sequencing errors. Deep sequencing was performed on an Illumina NovaSeq system using the PE150 mode. After error correction and deduplication, V, D, J, and C segments were mapped with NCBI, then CDR3 regions were extracted, and clonotypes were assembled.

#### 5.2.16 Permeability Assay

ISTR mice received tail vein injection of TRITC‐dextran (20 kDa MCE, HY‐158082A) at 5 µL/g body weight of 2 mg/mL at 3 d and 4 w post‐transplantation. 4‐week‐old BALB/c mice were used as controls. The tissues were collected 10 min later and immediately fixed at 4°C for 15 min before being embedded in O.C.T. and sliced. After a second fixation and DAPI staining, slices were observed on a Leica TCS SP8‐MP Multiphoton Microscope.

#### 5.2.17 Angiographic Analysis

The regenerated thymic integration was observed as described [50]. Briefly, cultured thymus tissue from GFP^+^ mice was transplanted into the spleen of nude mice. DyLight^TM^649 labeled lectin (VectorLabs, California, USA, DL‐1178‐1) was injected via the tail vein at various time points. After 10 min, the mice were anesthetized and perfused with PBS. The spleens were visualized using a Zeiss LSM 980 confocal microscope. Vascular networks were reconstructed with Imaris.

#### 5.2.18 Histology

Tissues were fixed in 4% paraformaldehyde and then embedded in paraffin after serial dehydration. For H&E staining and Masson's trichrome staining, sections (3–5 µm) were stained per manufacturer's protocols with H&E (Leagene, DH0001, DH0055) and Masson's trichrome (KeyGen Biotech, KGE1113‐8) staining kits.

For immunofluorescence, after gradient hydration, sections were heated for 15 min in Tris‐EDTA buffer (pH = 9.0, Solarbio, C1038) or improved citrate antigen‐retrieval solution (pH = 6.0, Beyotime, P0083) for antigen retrieval. Sections were blocked with 5% bovine serum albumin (BSA) (Beyotime, ST023) for 1 h, incubated with primary antibodies at 4°C overnight, and species‐matched secondary antibodies (1:200) at room temperature for 1 h at the appropriate dilution ratio. Slides were then stained with DAPI (Beyotime, C1002), mounted in mounting media (Beyotime, P0126), and imaged using the Zeiss LSM 980 confocal microscope.

Multiplex immunofluorescence staining was conducted via tyramide signal amplification (TSA). Briefly, paraffin‐embedded sections were dewaxed, rehydrated, and subjected to antigen retrieval. After 5% BSA blocking for 30 min, sections were incubated with the first primary antibody at 4°C overnight, washed in TBST, and treated with HRP‐linked secondary antibody for 1 h at room temperature. Fluorescent signals were developed with TSA fluorophore solution in the dark, followed by extensive TBST rinsing. Slides were reheated in retrieval buffer to strip bound antibodies and abolish residual HRP activity, then stained sequentially for the second and third targets using distinct TSA fluorophores. Sections were counterstained with DAPI, mounted with anti‐fade medium, and imaged under the microscope.

The primary antibodies included: Cytokeratin 5 (CK5) (Abcam, ab52635, 1:100), Cytokeratin 8 (CK8) (Abcam, ab53280, 1:500), CD3 (Abcam, ab16669; 1:200), epithelial cell adhesion molecule (EpCAM) (Proteintech, 21050‐1‐AP, 1:400), CD11c (Cell Signaling Technology, 97585S, 1:200), MHC‐II (Invitrogen, 14‐5321‐81, 1:500), AIRE (Abcam, ab243169, 1:500), Involucrin (Signalway Antibody, 33410, 1:100), claudin 5 (Abcam, ab131259, 1:500), collagen I (Abcam, ab270993; 1:1000), collagen IV (Abcam ab6586, 1:200), anti‐Endomucin (EMCN) (Invitrogen HM1008, 1:250), VSV‐G (Abcam, ab183497, 1:200), CD8 (Abcam, 228965, 1:200), CD19(Abcam, 134114,1:500), anti‐LCMV nucleoprotein antibody (VL‐4) (MCE, HY‐P990810, 1:200).

The secondary antibodies (Invitrogen, USA) were: anti‐rabbit Alexa Fluor 488, 546 and 647, anti‐rat Alexa Fluor 546 and 647; and anti‐mouse Alexa Fluor 546 and 647 secondary antibodies.

For H&E and Masson staining images, the NIKON DS‐RI2 microscope and the Olympus SLIDEVIEW VS200 Slide Scanner were used. Zeiss LSM 980 confocal microscope and Zen Black software were used to acquire immunofluorescence images. For statistical analysis, images were analyzed using Fiji and QuPath (v0.6.0) [67] software. The extent of histologic pathology was assessed based on a previous study [65].

#### 5.2.19 Flow Cytometry

Tissue dissociation: The spleens or lymph nodes were isolated and then placed on a 70 µm filter on top of a 6‐well plate with 5 mL precooled RPMI 1640 after the connective tissue was removed. The sample was sliced into small pieces, mashed through a strainer, and washed with medium until most of the tissue was dissociated. After washing and centrifugation, cells were subjected to erythrocyte lysis. For bone marrow, femurs and tibias were removed from the mouse and kept in cold RPMI 1640. The bones were placed in cold 10% FBS medium and are completely exposed with no remaining muscle fibers. Cells were isolated from cleaned femurs and tibias by crushing the bones in 10% FBS medium using a syringe plunger until the cell suspension is colorless. Cells were collected by filtration and centrifugation. The cell pellet was resuspended for erythrocyte lysis. Thymocytes were isolated from the thymic tissues by squeezing small pieces of thymic fragments through a filter. For isolation of thymic epithelium and immune cells, the thymus was minced and transferred to pre‐warmed digestion medium containing 1 mg/mL DNase I (Roche, 10104159001) and 0.5 mg/mL collagenase I (Nanjing Warbio Biotechnology, J1251) in RPMI 1640. Tissues were shaken vigorously for 30 s and further incubated at 37°C for 15–30 min with general shaking. Released cells passed through a 70 µm cell strainer (SPL Life Sciences, SPL‐93070) were washed, collected, and suspended in FACS buffer (HBSS with 1% BSA, Biosharp, BS114) for subsequent staining. Dissection of the muscle was done essentially as described [68]. Briefly, after removal of adipose and tendon, the quadriceps femoris was dissected, minced and digested in a digestion solution mix (0.5 mg/mL DNase, 1 mg/mL collagenase I, 1 mg/mL collagenase II (Nanjing Warbio Biotechnology, J1351) and 1.2 mg/mL dispase (Nanjing Warbio Biotechnology, J1621)) for 30–45 min at 37°C in a thermostatic shaker (90 r/min) mixed by inversion every 10 min. The digested muscle slurry was triturated briefly and then passed 10 times through a 20‐gauge needle syringe after 15 min of incubation, after which it was returned to the incubator. The homogenous solution was subsequently diluted with PBS containing 10% FBS and 2 mm EDTA, followed by filtration, and red blood cell lysis (TBDscience, NH4CL2009).

Single‐cell suspensions were stained with ad hoc antibody mixes (Table S1) diluted according to the manufacturer's suggestions in FACS buffer for 30 min on ice. Mouse FOXP3 was also intracellularly stained with the FOXP3/Transcription Factor Staining Buffer set (BioLegend) according to the manufacturer's instructions. For human intracellular cytokines or FOXP3 detection, splenic mononuclear cells were incubated in medium with 50 ng/mL PMA plus 1 µg/mL ionomycin mixture (MULTI SCIENCES, CS1001) or medium control for 4 h in the presence of 5 µg/mL brefeldin A (BD, 555029) before staining. Cells were intracellularly stained against IFN‐γ, IL‐17, IL‐4, FOXP3, CK5 and CK8 according to the manufacturer's instructions with an intracellular staining buffer kit (eBioscience, Cat# 00‐5523‐00). All samples were compared to fluorochrome‐matched IgG controls. 7‐AAD (BioLegend) or Zombie Live‐Dead dye (Zombie NIR Fixable Viability Kit, BioLegend) was used to discriminate live from dead cells.

Gating strategies are detailed in the Supporting Information. Cells were gated based on fluorescence minus one control. The absolute numbers of T, B, and CD45^+^ cells were calculated using flow cytometry frequencies and white blood cell values obtained from complete blood count (CBC) analysis. All corresponding FCM analyses were performed on FlowJo 10 (v.10.5.3) software.

#### 5.2.20 Lymphocyte Transformation Test

Freshly isolated lymphocytes were labeled with CFSE using a CFSE kit (KeyGEN BioTECH, KGA318) following the manufacturer's instructions and then cultured for 3 days in RPMI 1640/10% FBS medium with 5ug/mL concanavalin A (ConA) (Shanghai Yuanye Bio‐Technology, S12028) or medium alone. Cell proliferation was assessed by flow cytometry using CD45, CD3, CD4, and CD8 staining. Unstimulated and unlabeled T cells were used to set the gates.

#### 5.2.21 Magnetic Cell Sorting

CD4^+^ and CD8^+^ T cells were isolated from the spleen or lymph nodes (multiple anatomical sites) using MACS CD4 and CD8 magnetic microbeads (Miltenyi, Biotech) and negative selection columns (Miltenyi, Biotech) according to the manufacturer's instructions. The purity of sorted populations exceeded 90%.

#### 5.2.22 DNA Extraction and TREC Quantification

DNA from PBMC of aged‐matched nude, ISTR and BALB/c mice was isolated with Trizol and determined by NanoDrop Lite spectrophotometry. Relative expression of TREC levels to T cell receptor α constant (TCRA‐Cα) was quantified by qPCR using ChamQ SYBR mix on the StepOnePlus. Expression of TREC was normalized to that of the TCRA‐Cα and analyzed as 2^–ΔΔCt^. Each sample was tested in triplicate or more, and the experiment was independently repeated three times. Primers are in Table S2.

#### 5.2.23 Blood Routine Examination

Blood was obtained via tail vein puncture at various time points post‐operation. Blood samples were analyzed using an automated hematology analyzer (Mindray Animal Medical, BC‐5000 Vet) to determine various hematological parameters.

#### 5.2.24 ELISA

Coagulated blood samples collected from the mice's orbital venous plexus were centrifuged to obtain serum. ELISA kits were used according to the manufacturers’ instructions to measure the levels of serum IgM (Elabscience, E‐EL‐M0695c), IgG (Elabscience, E‐TSEL‐M0003), TNF‐α (eBioscience, 88‐7324‐77) and IFN‐γ (eBioscience, 88‐7314‐88) in the samples.

#### 5.2.25 ALT and AST Activity Assay

Serum ALT and AST activities were determined using Alanine Aminotransferase (ALT/GPT) Assay Kit (Nanjing Jiancheng, C009‐2) and Aspartate Aminotransferase (AST/GOT) Assay Kit (Nanjing Jiancheng, C010‐2) according to the manufacturers’ instructions. In brief, samples were incubated with substrate, followed by DNPH. The reaction was stopped with NaOH. The sample added after the reaction was used as a control. Absorbance was read at 505 nm (ALT) or 510 nm (AST). Activities (U/L) were calculated based on absolute OD values (Test—Control) and a standard curve.

#### 5.2.26 In Vivo Imaging

Ten days after VSV inoculation, mice were sacrificed after anesthesia, and tissues were excised and imaged for VSV‐GFP using the IVIS Spectrum In Vivo Imaging System (PerkinElmer). Pseudocolor images were generated by Living Image software.

#### 5.2.27 Cell Culture and Subcutaneous Xenograft Model

The tumor cells were maintained in 1640 or DMEM containing 10% FBS and penicillin and streptomycin under culture conditions (37°C, 5% CO_2_). 5×10^6^ B16, B16‐OVA, or HCT 116 cells suspended in 100 µL PBS were injected subcutaneously into the right flank of ISTR mice (8 weeks post‐graft), 12‐week‐old BALB/c nude or BALB/c mice (n = 5). B16 tumor weight was measured at 18 days post‐inoculation.

#### 5.2.28 ELISpot Assays

Splenocytes isolated from OVA‐B16 cells immunized mice (ST, 8 weeks post‐operation, nude, or BALB/c; n = 5) were seeded into IFN‐γ‐precoated strips (DAKEWE, 2210005) at 1 × 10^5^ per well with 50 µg/mL full‐length OVA protein or medium. After 20 h of incubation, the contents were decanted, and the cells were lysed in deionized water. The strips were washed six times between detection with biotinylated antibody, streptavidin‐HRP, and AEC substrate incubation. The reaction was stopped when distinct spots appeared. The membrane was dried and subjected to spot counting with the ImmunoSpot analyzer system.

#### 5.2.29 Hemoglobin Quantification

Hemoglobin levels were determined using the free hemoglobin assay kit (Nanjing Jiancheng, A071) according to the instructions. The tissue homogenate was diluted with cold PBS, mixed with a colorimetric reagent, and incubated at 37°C for 20 min. The absorbance of the solution was read at 510 nm against a reagent blank. All measurements were performed in triplicate. Absolute OD values (sample—blank) were converted to hemoglobin concentration (mg/dl) via a standard curve.

#### 5.2.30 RNA Sequencing and Differentially Expressed Genes Analysis

Total RNA of the spleen, muscle, and thymus from 4‐week‐old nude mice was extracted. RNA quality was evaluated using the NanoDrop (Thermo Scientific, USA) and the Agilent 2100 Bioanalyzer (Agilent Technologies, USA). The libraries were constructed using the VAHTS Universal V6 Kit and sequenced on the Illumina NovaSeq 6000 platform (OE Biotech, Inc., China). Differential gene expression analysis was performed using OECloud tools.

#### 5.2.31 GVHD Assessment

The severity of graft‐vs.‐host disease (GVHD) was monitored following the semi‐quantitative scoring system. Five independent clinical parameters were evaluated and graded from 0 (normal) to 2 (maximal severity): body weight loss, hunched posture, spontaneous activity, fur/skin integrity, and diarrhea. The sum of individual grades yielded a total clinical GVHD score ranging from 0 to 10 per mouse. Mice were humanely euthanized upon weight loss exceeding 30% of baseline body weight or when the total GVHD score reached ≥9, in accordance with institutional animal care and use committee (IACUC) guidelines.

#### 5.2.32 Statistical Analysis

Statistical analyses were performed with three or more biological replicates per group, with exact sample sizes (n) indicated. Normality was assessed before statistical comparisons. Normally distributed data were analyzed by unpaired two‐tailed *t*‐test, one‐way ANOVA, or two‐way ANOVA with Tukey's multiple‐comparison; non‐normal data were analyzed with the Mann–Whitney U test. *P* < 0.05 was considered significant. Data are presented as mean ± SEM. Graphs were generated using GraphPad Prism 10 and OriginPro 2023b.

## Author Contributions

Conceptualization: Lei Dong, Methodology: Shaocong Wang, Zijian Zhang, Fulin Dai, Zhijie Yin, Zhen Xing, Yurong Li, Chunyan Liu, Haochong Lei, Investigation: Lei Dong, Yan Li, Shaocong Wang, Junfeng Zhang, Visualization: Shaocong Wang, Lei Dong, Supervision: Lei Dong, Writing – original draft: Lei Dong, Shaocong Wang, Writing – review and editing: Lei Dong, Yan Li, Chunyan Liu, Haochong Lei.

## Funding

The National Natural Science Foundation of China grant 32230056 (L.D.), the National Natural Science Foundation of China grant 32361163656 (L.D.), the National Natural Science Foundation of China grant 32471000 (Y.L.), the National Natural Science Foundation of China grant U24A20378 (Y.L., J.Z.), the National Natural Science Foundation of China grant 32230058 (J.Z.), the National Science and Technology Major Program grant 2023ZD0500400 (Y.L.), the National Science and Technology Major Program grant 2025ZD01900700 (Y.L.), the Fundamental Research Funds for the Central Universities grant 2024300408 (Y.L.), the Fundamental Research Funds for the Central Universities grant XJ2024003602 (Y.L.), the Fundamental Research Funds for the Central Universities grant KG202511 (L.D.), the Jiangsu Provincial Science and Technology Plan Special Fund grant BK20243041 (L.D.), the Jiangsu Provincial Science and Technology Plan Special Fund grant BZ2024052 (L.D., J.Z.), the Fundamental and Interdisciplinary Disciplines Breakthrough Plan of the Ministry of Education of China grant JYB2025XDXM507 (L.D.).

## Conflicts of Interest

The authors declare no conflicts of interest.

## Supporting information




**Supporting File**: advs77358‐sup‐0001‐SuppMat.docx.

## Data Availability

Sequencing data have been deposited in the Gene Expression Omnibus (accession numbers: GSE310787 and GSE336449). The other datasets supporting the findings of this study are available from the corresponding author upon reasonable request.
