# Supplementary material for: Intrasplenic Thymus Organogenesis from Injectable Tissue Fragments Restores Functional T‐Cell Immunity
Source: Adv Sci (Weinh). 2026 Aug 25;13(63):e77358. doi: 10.1002/advs.77358 (PMC13505467; doi:10.1002/advs.77358)
Supplement: Supplementary file 1 [file ADVS-13-e77358-s001.docx]

**Supporting Information**

**Intrasplenic Thymus Organogenesis from Injectable Tissue Fragments Restores Functional T-cell Immunity**

**Authors**

Shaocong Wang^1†^, Zijian Zhang^2†^, Fulin Dai^1^, Zhijie Yin^1^, Zhen Xing^1,3^, Yurong Li^1,4^, Haochong Lei^6^, Junfeng Zhang1, Chunyan Liu^1*^, Yan Li^2,3,5*^, and Lei Dong^1,2,3,5*^

**Affiliations**

^1^State Key Laboratory of Pharmaceutical Biotechnology, School of Life Sciences, Nanjing University, Nanjing, Jiangsu, China.

^2^National Resource Center for Mutant Mice, MOE Key Laboratory of Model Animal for Disease Study, Model Animal Research Center, Department of Oncology, Nanjing Drum Tower Hospital, Affiliated Hospital of Medical School, Nanjing University, Nanjing, China.

^3^Wuxi Xishan Institute of Applied Biotechnology, Nanjing University, Wuxi, Jiangsu, China.

^4^Jiangsu Key Laboratory of Sericultural Biology and Biotechnology, School of Biotechnology, Jiangsu University of Science and Technology, Zhenjiang, Jiangsu, China.

^5^Chemistry and Biomedicine Innovation Center (ChemBIC), ChemBioMed Interdisciplinary Research Center at Nanjing University, Nanjing, Jiangsu, China.

^6^Faculty of Life Sciences & Medicine, King’s College London, London, United Kingdom.

† These authors contributed equally to this work.

***** Corresponding author. Email: leidong@nju.edu.cn (L. D.), yanli@nju.edu.cn (Y. L.), cyliu@nju.edu.cn (C. L.)

**Materials and Methods**

**Indexes of TCR diversity**

Shannon’s index is used to measure the diversity of TCR clones. It can describe two aspects of information: (1) species richness; (2) evenness of individual distribution in species. The higher the Shannon index, the higher the diversity of TCR.

The entropy H’ was calculated according to the following formula:

$$H'=-\sum pi\left(\ln pi\right)$$

where “pi” represents the frequency of the specific clone type of “i” in the sample.

The clonality index measures the degree of TCR/BCR clonal expansion, where higher clonality values indicate that specific clones have undergone high-frequency expansion. The Clonality was calculated as:

$$Clonality=1-\frac{Diversity}{\ln\left(productiveuniques\right)}$$

In the formula, productiveuniques denotes the total number of distinct clonotypes in the sample. Diversity corresponds to the Shannon diversity index value.

Pielou's index is used to evaluate the uniformity of clonal distribution. A higher Pielou's value indicates a more even distribution across clones, while a lower value suggests expansion of specific clonal populations. Pielou’s was computed as:

$$Pielou's=-\sum\frac{pi\left(\ln pi\right)}{\ln S}$$

In the formula, “pᵢ” represents the proportion of the i-th sequence, and “S” denotes the total number of clonal types.

The Inverse Simpson index (reciprocal of the Simpson index) quantifies the probability that two randomly selected clonotypes from a repertoire belong to different clonal types. A larger inverse Simpson value reflects higher diversity, particularly emphasizing the contribution of high-frequency clonotypes.

The formula is defined as:

$$\frac{1}{D}=\frac{1}{\sum ni\left(ni-1\right)/N\left(N-1\right)}$$

where “ni” represents the total number of amino acid sequences for the i-th specific clonotype, and “N” represents the total number of sequences in the sample.

The Hvj diversity index quantifies V-J gene pairing diversity in TCR/BCR repertoires. Elevated values indicate antigen-driven clonal expansion of T or B lymphocytes. The entropy H’ was computed as:

$$H'=-\sum pi\left(\ln pi\right)$$

where “pi” denotes the proportion of the i-th V-J pairing fragment type.

DE50 serves as an indicator of clonality, reflecting the uniformity of the clonal population. The higher the value, the more evenly clonal the distribution; conversely, the lower the DE50 value, the more likely specific clones have undergone amplification.

The calculation method of the formula is:

where “n” represents the number of top clones that account for more than 50% of the total, and “N” denotes the total number of clones within the clone group.

**Figure S1. Examination of cultured thymus tissue and optimization of tissue size for intrasplenic thymus transplantation.** (**A**) Representative macroscopic images of fresh-thymus-implanted and control nude mice. Scale bar: 1 cm. (**B**) H&E staining of thymic tissue before and after culture at low and high magnification. Scale bar: 100 µm, 50 µm. (**C** and **D**) Proportions of CK5^+^ or CK8^+^ single positive (SP, left) epithelial cells in EpCAM^+^ epithelial cells and CK5^+^CK8^+^ double positive (DP, right) in DP + SP population (C) and gating strategy (D). Gating sample: Fresh thymus from P0 newborn mice. (**E**) Immunofluorescence staining of CD3 and EpCAM in uncultured and cultured thymic tissue. Images of unstained control under the same conditions are shown (right). Scale bar: 20 µm. (**F**) Gating strategy for flow cytometry analysis of thymic tissue. The sample used for gating was tissue cultured for 4 days. (**G**) Cellular composition of CD3^+^ thymocytes, B cells and DCs in thymic tissue before and after culture. (**H** and **I**) Morphology (H) and weight (I) of thymic tissue with different diameters (0.2, 0.5, 0.7 and 0.9 mm) at week 4 post-transplantation (n = 5). Scale bars: 200 µm, 50 µm. Data are represented as mean ± SEM. Statistical analysis was performed using one-way ANOVA. **P* < 0.05, *****P* < 0.0001, n.s., not significant. All experiments were repeated twice per group.

**Figure S2. Fluorescence images of intramuscular thymic tissue at various time points.** The quadriceps femoris tissue was exposed to GFP excitation, and GFP^+^ thymic tissue images were collected at different time points using the corresponding filter. Scale bar: 2 mm.

**Figure S3.** **Superiority conditions of the spleen for thymus growth.** (**A**) Hemoglobin levels in the spleen and muscle tissue (n = 5). (**B**) Vascularization of thymic tissue in the spleen and the muscle at week 2 post-graft. Scale bar: 100 μm. (**C** and **D**) Transcriptome analysis of the spleen and muscle tissue (n = 3). (C) Heatmap depicting the expression of thymus-regeneration-promoting factors. Rows: gene name; Columns: muscle (green), thymus (orange) and spleen (blue); color bar: blue-white-red scale represents log_2_(FPKM) from -2 to 2. (D) Volcano plot displaying differentially expressed genes. Gray: non-significant; Red: upregulated (log2FC > 1, *p < 0.05*); Blue: downregulated (log2FC <-1, p < 0.05). Interested genes are labeled. (**E**) The classical pathways of T-cell differentiation. HSCs differentiate into LMPP or CLP before entering the thymus, and subsequently develop into T cells. (**F**) Gating strategy for CLP (red) and LMPP (blue) in bone marrow samples by flow cytometry. (**G**) Proportions of CLP, LMPP(IL-7R^-^), LMPP(IL-7R^+^), total LMPP and CLP + LMPP in the thymus, spleen and muscle (n = 5). Data are represented as mean ± SEM. Statistical analysis was performed using an unpaired two-tailed Student's t test (A) and one-way ANOVA (G). **P* < 0.05, *****P* < 0.0001, n.s., not significant. All experiments were independently repeated twice per group, except for panels (C) and (D).

**Figure S4.** **Development of thymic tissue in the spleen.** (**A**) Immunofluorescent staining of CK8 and endomucin of intrasplenic thymic tissue at various time points. Scale bar: 50 μm. (**B**) Confocal 3D imaging (i) and surface modeling (ii) of the integration of splenic circulation (labeled with DyLight^TM^ 649 Lycopersicon esculentum lectin) and EGFP-labeled thymus grafts to monitor vascularization after transplantation. Scale bar: 50 µm, 80 µm. (**C** and **D**) Immunofluorescent staining for medulla-specific markers, (C) CD11c (DCs) and MHC-II, (D) AIRE and involucrin (Hassall's corpuscle) at week 4 post-graft. Scale bar: 100 µm, 20 µm. (**E**) Relative expression of thymus functional genes within BALB/c (8-week-old) and ISTR (8 weeks post graft), (n = 3). Data are mean ± SEM. Statistical significance was determined by Student’s *t*-test (E); **P* < 0.05, *****P* < 0.0001; n.s., not significant. All experiments were independently repeated twice.

**Figure S5. Restoration of the blood-thymus barrier.** (**A**) TRITC-dextran permeability of EGFP-labeled thymus tissue at day 3 and week 4 post-graft in frozen sections. Dotted lines indicate borders between the cortex (C) and medulla (M), defined by DAPI staining. Scale bar: 100 μm. (**B**) Masson staining (i) and immunofluorescent staining of CK8, collagen I and collagen IV (ii) displayed the barrier of thymic tissue in the spleen. Scale bar: 100 μm. (**C**) Immunofluorescent staining for the colocalization of endomucin and claudin-5 in the cortical areas (CK8^+^) of intrasplenic thymic tissue, in situ thymus of 4-week-old BALB/c mice, was used as control. Scale bar: 20 μm. The experiments were repeated 3 times for each group.

**Figure S6.** **Hematological analysis of mice following sham operation and gating strategies for T-cell subsets.** (**A**) Hematological analysis of nude mice with sham-operation and untreated nude mice at week 2 post-operation (n = 7). (**B**) Spleen index with or without sham surgery in nude mice (n=6). (**C**) Gating strategy for CD4^+^ and CD8^+^ naive T cells. Gating sample: PB from ISTR mice 8 weeks post-transplantation. Data are mean ± SEM. Statistical significance was determined by Student’s t-test [(A) and (B)]; **P* < 0.05, *****P* < 0.0001; n.s., not significant. All experiments were independently repeated twice.

**Figure S7. Splenic engraftment supports faster and more robust thymic regeneration than intramuscular grafts.** (**A**) Peripheral blood leukocyte counts and percentages at week 8 in nude (gray), ISTR (red), IMTI (purple), and BALB/c (blue) mice (n = 7). (**B** and **C**) Week 8 absolute numbers and percentages of CD3⁺ T cells (B), and proportions of CD4⁺ and CD8⁺ T-cell subsets (C) in each group (n = 7). (**D**) Frequencies of naive (CD62L⁺CD44⁻) CD4⁺ and CD8⁺ T cells in peripheral blood at week 8 (n = 7). Data are mean ± SEM. Statistical comparisons by one-way ANOVA [(A) to (D)]; **P* < 0.05, *****P* < 0.0001; n.s., not significant. All experiments were independently repeated twice per group.

**Figure S8. Illustration of the subpopulations of non‑T lymphocytes in PBL.** Representative **s**equential gating of B cells, αβT cells, γδT cells, NK cells, neutrophils, macrophages and monocytes in the PBL of nude (12-week-old), ISTR (8 weeks post-graft) and BALB/c (12-week-old) mice. Experiments were independently repeated three times per group.

**Figure S9.** **Analysis of Treg cell populations.** (**A**) Representative flow cytometry plots showing Treg subsets within splenic CD4^+^ T cells from 12-week-old nude mice, ISTR mice at 8 weeks post-transplantation, and 12-week-old BALB/c mice. (**B**) Gating strategy for splenic Tregs in ISTR at 8 weeks post-transplantation. Experiments were independently repeated three times per group.

**Figure S10.** **Gating strategy for thymic CD4^+^CD8^+^ double-positive cells.** BALB/c thymus at the age of 8 weeks was used.

**Figure S11.** **Functional verification of T cell subsets.** (**A**) The lymphocyte transformation test was performed by CFSE dilution of T cells after 3 days of Con A stimulation. Representative flow cytometry plots for unstimulated vs. stimulated T-cell populations are shown. (**B**) T-cell stimulation index based on CFSE dilution (n = 6). (**C**)ELISPOT assay timeline: B16-OVA cells were injected on day 0; on day 7, splenocytes were co-cultured with OVA full-length protein for 20 h. (**D**) Representative IFN-γ ELISPOT wells. (**E**) Quantification of IFN-γ-secreting cells per 10^5^ responders (n = 5). Data are represented as mean ± SEM. Statistical analysis was performed using one-way ANOVA [(B) and (E)]. **P* < 0.05, *****P* < 0.0001, n.s., not significant.

**Figure S12.** **Antigen-restricted responses of CD8^+^ T cells.** Representative IFN-γ^+^ T cells within the CD8^+^ T-cell subset were shown in BALB/c mock or BALB/c OVA-preimmunized mice, as well as ISTR mock or ISTR OVA-preimmunized mice. Purified T cells were co-cultured with H2K^b^ or H2K^d^ restricted DCs loaded with or without full-length OVA protein for 16 h and analyzed by flow cytometry. Experiments were independently repeated twice per group.

**Figure S13.** **Antigen-restricted responses of CD4^+^ T cells.** Representative TNF-α^+^ T cells within the CD4^+^ T-cell subset were shown in BALB/c mock or BALB/c OVA-preimmunized mice, as well as ISTR mock or ISTR OVA-preimmunized mice. Purified T cells were co-cultured with H2K^b^- or H2K^d^-restricted DCs loaded with or without full-length OVA protein for 16 h and analyzed by flow cytometry. Experiments were independently repeated twice per group.

**Figure S14.** **Safety of the intrasplenic thymus transplantation technique.** (**A**) Gross overview of the nude mice (top) and ISTR mice (bottom) at week 12 post-graft. Scale bar: 5 mm. (**B**) Gross morphology of the main organs that are prone to GVHD. Scale bar: 5 mm. (**C**) Histomorphological analysis of the heart, liver, lung, kidney, colon and uterus of nude mice and ISTR mice. Scale bar: 100 µm in heart, liver, lung, kidney, and colon; 200 µm in uterus. (**D** to **F**) Assessment of inflammatory cytokines (TNF-α, n = 6; IFN-γ, n = 3) (D), total IgM and IgG (n = 5) (E), and ALT and AST (nude group, n = 5, ISTR and BALB/c groups, n = 9) (F) in the serum of ISTR mice at week 12 post-transplantation, samples from nude and BALB/c mice were used as controls. (**G**) Representative gross overview of the nude mice (left, n = 8), ISTR mice (middle, n = 12) and IMTI mice (right, n = 13) at week 12 post-graft. Scale bar: 1 cm. (**H**) Body weight of nude, ISTR, and IMTI mice at week 12 post-graft. (**I**) GVHD total score for nude, ISTR, and IMTI mice at week 12 post-graft. Data are represented as mean ± SEM. Statistical analysis was performed using one-way ANOVA for panels (D) to (F) and (H), and Student’s t-test for (I). **P* < 0.05, *****P* < 0.0001, n.s., not significant. Experiments were independently repeated three times per group in A-C.

**Figure S15. Long-term evaluation of peripheral T-cell profiles and chronic GVHD of ISTR mice.** (**A**) Representative overall macroscopic photographs of 11-month-old nude and ISTR mice. Scale bar: 1 cm. (**B**) Total GVHD score of 11-month-old nude, ISTR and BALB/c mice (nude mice: n =3, other groups: n = 4). (**C**) Proportion of CD3^+^ T cells in peripheral blood leukocytes. (**D**) Proportion of CD4^+^ and CD8^+^ T-cell subsets in PBL T cells. (**E**) Proportion of Treg subsets in PBL T cells. (**F**-**G**) Proportion of PD-1^+^(F) and CD69^+^(G) T cells within CD4^+^ or CD8^+^ subsets. (**H**) Representative H&E-stained images of the spleen and liver from 11-month-old nude, ISTR, and BALB/c mice. Scale bar: 100 μm. (**I**) Representative Masson staining images of the liver, lung, and skin of 11-month-old nude, ISTR, and BALB/c mice. Scale bar: 100 μm. Data are represented as mean ± SEM. Statistical analysis was performed using one-way ANOVA for panels (C) to (G). **P* < 0.05, *****P* < 0.0001, n.s., not significant. Experiments were independently repeated twice per group.

**Figure S16. Cellularity of the heterotopic thymus graft of aged mice.** Total thymocytes, as well as DN, DP, CD4^+^SP, and CD8^+^SP thymocytes, from the intrasplenic or intramuscular grafts at week 10 post-transplantation in old mice were measured by flow cytometric analysis (n = 5). Data are mean ± SEM. Statistical significance was determined by Student’s *t*-test; **P* < 0.05, *****P* < 0.0001; n.s., not significant.

**Figure S17. Allogeneic hematopoietic stem cell transplantation in thymectomized BALB/c mice.** (**A**) Experimental scheme of the HSCT timeline. 6-week-old female BALB/c mice were thymectomized 7 days prior to lethal irradiation (8 Gy). HSCs (for group Thx.IRR.HSC) or T-cell-depleted HSCs were isolated from the bone marrow of 6–8-week-old female C57BL/6J-GFP^+^ mice, and were infused into the thymectomized recipients through the tail vein. Cultured thymic tissue was introduced into the spleen (group Thx.IRR.TCD-HSC.ISTR) or quadriceps femoris (group Thx.IRR.TCD-HSC.IMTI) of T-cell-depleted HSC-adopted mice. Mice receiving T-cell-depleted HSCs without thymus implantation served as controls (group Thx.IRR.TCD-HSC). (**B**) Flow cytometric analysis showing T cell profiles of BM derived HSCs and T-cell-depleted HSCs.

**Figure S18. Antivirus responses of ISTR mice.** (**A** and **B**) Representative images of the spleens (A) and spleen index (n = 5) at day 10 post-infection (B). (**C**) Representative H&E staining of small intestine sections at day 10 post-infection. Brown arrow: increased lamina propria cellularity; black arrow: denuded villi tip. Scale bar: 200 μm, 100 μm. (**D**) Representative immunofluorescence images of LCMV nucleoprotein in lymph nodes collected at day 12 post-infection. Scale bar: 100 μm. Data are represented as mean ± SEM. Statistical analysis was performed using one-way ANOVA (B). **P* < 0.05, *****P* < 0.0001, n.s., not significant. Experiments were independently repeated three times per group in C and D.

**Figure S19.** **Antitumor responses of ISTR mice.** (**A** and **B**) Subcutaneous challenge with B16 melanoma cells; tumor weights measured on day 18 (n = 5). (**C**) Representative H&E staining and immunofluorescence staining of CD3^+^ T-cell and CD8^+^ T-cell infiltration of B16 tumors. Scale bar: 100 µm, 50 µm. (**D** and **E**) Subcutaneous inoculation with HCT116 colorectal cells and gross appearance of HCT116 tumors 18 days after implantation; tumor margins indicated by dashed lines. (**F**) Tumor take rate in nude, ISTR, and BALB/c mice (n = 10). Data are represented as mean ± SEM. Statistical analysis was performed using one-way ANOVA (B). **P* < 0.05, *****P* < 0.0001, n.s., not significant. All experiments were independently repeated twice for each group.

**Figure S20. Recovery of the thymic tissue after sublethal irradiation.** (**A**) Experiment scheme: cultured thymus was transplanted into the spleen or quadriceps femoris of the nude mice 6 weeks prior to sublethal X-ray irradiation (3 Gy), blood and thymic grafts were collected at days 7, 14, and 35, respectively, to evaluate the recovery of the T cell output and thymic graft cellularity. (**B** and **C**) Weight (B) and cellularity (C) of grafts isolated from the spleen or quadriceps femoris at days 7, 14, and 35 post-irradiation, respectively. (**D** and **E**) Thymocyte composition (D) and DN thymocyte subset ratio (E) of untreated or irradiated BALB/c thymus, or irradiated thymus grafts from the spleen or quadriceps femoris at days 7, 14, and 35 post-irradiation, respectively. (**F**) Naive T-cell counts and the proportion of naive T cells in PBL across experimental groups on days 7, 14, and 35 post-irradiation, respectively. (**G**) H&E staining revealing the intrasplenic or intramuscular graft morphology at days 7, 14, and 35 post-irradiation, respectively. Scale bar: 100 μm. Data are represented as mean ± SEM. Statistical analysis was performed using Student’s *t*-test (B) ,(C), and (F). **P* < 0.05, *****P* < 0.0001, n.s., not significant.

**Figure S21. Performances of the second transplanted grafts.** (**A**) Experimental scheme: primary thymic grafts were isolated from the spleen or quadriceps femoris at week 6 after the first transplantation, and were transplanted into the same position as the primary transplantation in new 4-week-old nude mice. Blood and spleen were analyzed at 6 weeks post the second transplantation. (**B**) Proportion of T cells in the PBL and splenocytes (n = 4). (**C**) Graft diameter of the intrasplenic or intramuscular thymus tissue before second transplantation and 6 weeks post-second transplantation. Data are represented as mean ± SEM. Statistical analysis was performed using one-way ANOVA [(B) and (C)]. **P* < 0.05, *****P* < 0.0001, n.s., not significant.

**Figure S22.** **Composition of human immune cells and human T cells in humanized mice.** (**A**) Flow cytometry plot of human T cells in peripheral blood of NCG-X and IST mice (intrasplenic thymus transplantation only) at week 13 post-graft (n = 3). (**B**) Representative flow cytometry plots of human T cell proportion at week 13 post-HSC transplantation (For the HSC group, n = 3; for the HSC + IST and HSC + IMT groups, n = 5). (**C** to **F**) Gating strategy for human T cells, CD4^+^ and CD8^+^ T cells (C), CD45^+^ cells (D), B cells (E), and naive T cells (F). (**G**) Representative analysis of T-cell activation from HSC + IST group after stimulation with PMA + ionomycin. All experiments were independently repeated twice.

**Table S1. Antibody List in Flow Cytometry Analysis**

| Antibody | Brand Name | Catalog number |
| --- | --- | --- |
| FcR Blocking Reagent, mouse | MACS | 130-092-575 |
| FITC anti-mouse CD326 (Ep-CAM) | Biolegend | 118207 |
| PE anti-mouse CD3ε | Biolegend | 100307 |
| APC anti-mouse CD19 | Biolegend | 115512 |
| APC anti-mouse CD11c | Biolegend | 117310 |
| APC/Cyanine7 anti-mouse CD45 | Biolegend | 103116 |
| FITC anti-mouse CD45 | Biolegend | 103018 |
| Brilliant Violet 711 anti-mouse CD3 | Biolegend | 100241 |
| Brilliant Violet 421 anti-mouse CD4 | Biolegend | 100443 |
| FITC anti-mouse CD4 | Biolegend | 100406 |
| APC anti-mouse CD8a | Biolegend | 100712 |
| PE anti-mouse CD8a | Biolegend | 100708 |
| PE/Cyanine7 anti-mouse/human CD44 | Biolegend | 103030 |
| APC anti-mouse CD62L | Biolegend | 104412 |
| APC anti-mouse CD25 | Biolegend | 102012 |
| PE anti-mouse FoxP3 | Biolegend | 126404 |
| FITC anti-mouse CD69 | Biolegend | 104505 |
| Biotin anti-mouse Lineage Panel | Biolegend | 133307 |
| FITC Avidin | Biolegend | 405101 |
| PE/Cyanine7 anti-mouse CD117 (c-kit) | Biolegend | 135111 |
| Brilliant Violet 421™ anti-mouse CD135 | Biolegend | 135313 |
| PE anti-mouse CD127 (IL-7Rα) | Biolegend | 158203 |
| APC anti-mouse Ly-6A/E (Sca-1) | Biolegend | 108111 |
| FITC anti-human CD45 | Biolegend | 304006 |
| APC anti-human CD3 | Biolegend | 300412 |
| PE anti-human CD8a | Biolegend | 301008 |
| PE anti-human CD19 | Biolegend | 302208 |
| FITC anti-human CD45RA | Biolegend | 304105 |
| PE anti-human CD45RO | Biolegend | 376803 |
| Spark Red™ 718 anti-human CD4 | Biolegend | 344688 |
| PE/Cyanine7 anti-human CD62L | Biolegend | 304821 |
| BV605 Mouse Anti-Human CD4 | BD | 562843 |
| APC-cy7 anti-human CD3 | Biolegend | 300426 |
| PE-cy5 anti-human CD45 | Ebioscience | 15-0459-42 |
| PE-cf594 anti-human CD8 | Biolegend | 301058 |
| Percp-cy5.5 anti-human IFN-y | Biolegend | 502526 |
| PE-cy7 anti-human IL-4 | Ebioscience | 25-7049-82 |
| AF700 anti-human Foxp3 | Ebioscience | 56-4776-41 |
| BV785 anti-human IL-17a | Biolegend | 512338 |
| Percp-cy5.5 anti-mouse CD8α antibody | eBioscience | 45-0081-82 |
| APC anti-mouse/human Foxp3 antibody | eBioscience | 50-4774-42 |
| AF700 anti-mouse CD69 antibody | eBioscience | 56-0691-82 |
| APC-cy7 anti-mouse CD4 antibody | eBioscience | 47-0042-82 |
| BV605 anti-mouse PD-1 antibody | Biolegnd | 135220 |
| PE anti-mouse CD3ε antibody | BD | 553240 |
| PE-cy7 anti-mouse CD45 antibody | Biolegend | 103114 |
| PE-CF594 anti-CD11b antibody | BD | 562287 |
| APC anti-mouse CD45 antibody | Biolegend | 103112 |
| AF700 anti-mouse CD3ε antibody | Biolegend | 152316 |
| PE anti-mouse IFNγ antibody | Biolegend | 163504 |
| PE-eFluor 610 anti-mouse TNFα antibody | eBioscience | 61-7321-82 |
| FITC anti-mouse/human B220 antibody | Biolegend | 103205 |
| Percp-cy5.5 anti-mouse Ly6G antibody | BD | 560602 |
| APC anti-mouse NKP46 antibody | Biolegend | 325112 |
| BV650 anti-mouse TCR γ/δ antibody | Biolegend | 118147 |
| BV785 anti-mouse Ly6C antibody | Biolegend | 128041 |
| PE/Dazzle594 anti-mouse CD24 Antibody | Biolegend | 101838 |
| BV421 anti-mouse CD25 antibody | BD | 564370 |
| BUV737 Hamster Anti-Mouse TCR β Chain | BD | 612821 |
| PE anti-mouse KLRG1 antibody | Biolegend | 164904 |
| PE anti-mouse CD326 (Ep-CAM) | BioLegend | 118205 |
| PE/Cyanine7 anti-mouse IgG1 | BioLegend | 406613 |
| CK5 Antibody | Affinity | BF0493 |
| CK8 Antibody | Abcam | ab53280 |

**Table S2. Primers List**

| Gene Name | Forward primer | Reverse Primer |
| --- | --- | --- |
| mus-sjTREC | CAAGCTGACAGGGCAGGTTT | TGAGCATGGCAAGCAGTACC |
| mus-TCRA-Ca | TGACTCCCAAATCAATGTG | GCAGGTGAAGCTTGTCTG |
| Mus-Dll4 | CAGTTATGCCTGCGAATGCC | TTCGGCTTGGACCTCTGTTC |
| Mus-Dll1 | TGGGCTTCTCTGGCTTCAAC | GGTAAGAGTTGCCGAGGTCC |
| Mus-Ccl25 | AAGACTGCTGCCTGGGTTAC | GCTTCCACTCACTTCCTGCT |
| Mus-Il7 | TGCTGCCTGTCACATCATCT | GTTTGGTTCATTATTCGGGCA |
| Mus-Foxn1 | GTGGTCCTGGGATGTACTGC | AGGGATAGTGAGGTGAGCCC |
